# A New Generator of Probability Models: The Exponentiated Sine-G Family for Lifetime Studies

**DOI:** 10.3390/e23111394

**Published:** 2021-10-24

**Authors:** Mustapha Muhammad, Huda M. Alshanbari, Ayed R. A. Alanzi, Lixia Liu, Waqas Sami, Christophe Chesneau, Farrukh Jamal

**Affiliations:** 1 School of Mathematical Sciences, Hebei Normal University, Shijiazhuang 050024, China; mmmahmoud12@sci.just.edu.jo; 2 Department of Mathematical Sciences, Bayero University, Kano 700241, Nigeria; 3 Department of Mathematical Sciences, College of Science, Princess Nourah Bint Abdulrahman University, P.O. Box 84428, Riyadh 11671, Saudi Arabia; hmalshanbari@pnu.edu.sa; 4 Department of Mathematics, College of Science and Human Studies at Hotat Sudair, Majmaah University, Majmaah 11952, Saudia Arabia; a.alanzi@mu.edu.sa; 5 Department of Mathematics, College of Science and Arts in Gurayat, Jouf University, Gurayat 77454, Saudi Arabia; 6 Department of Community Medicine & Public Health, College of Medicine, Majmaah University, Almajmaah 11952, Saudi Arabia; w.mahmood@mu.edu.sa; 7 Azra Naheed Medical College, Superior University, Lahore 54000, Pakistan; 8 Department of Mathematics, University of Caen-Normandie, 14032 Caen, France; christophe.chesneau@unicaen.fr; 9 Department of Statistics, The Islamia University of Bahawalpur, Punjab 63100, Pakistan

**Keywords:** sine-generated family, Weibull distribution, quantile, entropy, parametric estimation, Bayes estimation, stress-strength reliability

## Abstract

In this article, we propose the exponentiated sine-generated family of distributions. Some important properties are demonstrated, such as the series representation of the probability density function, quantile function, moments, stress-strength reliability, and Rényi entropy. A particular member, called the exponentiated sine Weibull distribution, is highlighted; we analyze its skewness and kurtosis, moments, quantile function, residual mean and reversed mean residual life functions, order statistics, and extreme value distributions. Maximum likelihood estimation and Bayes estimation under the square error loss function are considered. Simulation studies are used to assess the techniques, and their performance gives satisfactory results as discussed by the mean square error, confidence intervals, and coverage probabilities of the estimates. The stress-strength reliability parameter of the exponentiated sine Weibull model is derived and estimated by the maximum likelihood estimation method. Also, nonparametric bootstrap techniques are used to approximate the confidence interval of the reliability parameter. A simulation is conducted to examine the mean square error, standard deviations, confidence intervals, and coverage probabilities of the reliability parameter. Finally, three real applications of the exponentiated sine Weibull model are provided. One of them considers stress-strength data.

## 1. Introduction

In statistics, probability models are essential tools for modeling random phenomena. New flexible models were presented and examined in the literature in recent decades. This allowed a more deep exploration of real-life phenomena. The advancement of research in numerous domains, such as reliability, survival analysis, computer sciences, finance, biomedical sciences, medicine, hydrology, etc., leads researchers to propose more flexible distribution for better modeling of various kinds of data experience in practical applications. Most of the probability distributions were proposed based on algebraic functions. Since the von Mises (VM) distribution (see [1]), circular Cauchy (CC) distribution (see [2]), and Fisher distribution (see [3]), etc. However, there are very few distributions involving the so-called trigonometric functions. The recent ones include the beta-trigonometric (BT) distribution (see [4]), sine square (SS) distribution (see [5]), hyperbolic cosine-F (HC-F) distribution (see [6]), and distributions of the sine-generated (SG) family (see [7]). Further, ref. [8] introduced the cosine-sine-generated (CSG) family, ref. [9] provided the new-sine-generated (NSG) distribution and highlighted some of the advantages of sine-generated distributions in statistical studies, ref. [10] proposed the polyno-expo-trigonometric (PET) distribution, ref. [11] elaborated the new exponential with trigonometric function (NET), and [12] created the weighted-cosine exponential distribution. Due to the increase in interest in data analysis, researchers are required to go further to have more choices in terms of the availability of distributions that focus on trigonometric functions.

On the other hand, over the past decades, probability generators have greatly contributed to distribution theory, leading to several important mathematical and statistical tools useful in both theory and practice. Thus, we believe that involving trigonometric functions in distribution theory through various viewpoints can solve several complex problems in the future concerning the theoretical aspects and practice in various fields of study. The purpose of this study is to provide another new flexible probability model/distribution generator, called the exponentiated SG (ESG) family and provide several useful theoretical and practical results. In addition, the modeling capabilities of the SG family in practice draw our attention to further investigation and exploration of the SG family in its generalized form. The ESG family can be considered as a natural one-parameter extension of the SG family and an alternative to many other power-exponential-logarithmic distributions. It is defined by a cumulative distribution function (CDF) expressed as the power of the CDF of the SG family. The additional tuning power parameter allows us to modulate the functionalities, including the oscillatory amplitude, of the CDF of the SG family, opening some new statistical perspectives for data fitting, among other things. Surprisingly, this very intuitive concept did not appear to receive much attention in the literature. We investigate the fundamental properties in a closed-form and real-world applications of the ESG family, as well as some computational algorithms and techniques. Different distributions serve different purposes and represent different data generation processes. The ESG strategy provides other means of generating new probability models containing some trigonometric functions. The exponentiated sine-Weibull (ESW) distribution is proposed as a particular member of the ESG family and expresses closed-form expressions of several properties and statistical measures, such as the moments, asymptotic residual life, and distributions of extreme values. By presenting an estimation method and a real-world application of the ESW distribution, the potential and applicability of the new family in the stress strength reliability analysis are discussed. On the other hand, most statistical properties of distributions derived using trigonometric functions raised to a certain power are not fully expressed analytically in the literature; here, we are able to express the series representation of the sine and cosine functions to the power of a real number, which hopefully will be useful. In addition, a result by [13] provides differentiation formulas and power expansions for trigonometric functions to the power of positive integers and summation formulas. We hope that these series representations will be the means of extending several mathematical results in applied mathematics.

The following is the plan of the paper: In Section 2, the ESG family is specified. Some of its key characteristics are discussed. In Section 3, the expression of a reliability parameter is derived. In Section 4, the ESW distribution is defined. A few of its most important properties are discussed. In Section 5, maximum likelihood estimation and Bayes estimation for the ESW distribution are proposed and examined by simulation studies. Among other things, the maximum likelihood estimation of the reliability parameter of the ESW model is obtained. The nonparametric percentile bootstrap and Student’s bootstrap are considered to approximate the reliability parameter confidence interval, also discussed in simulation studies. Three examples of application to real data sets can be found in Section 6. In Section 7, conclusions are suggested.

## 2. The Exponentiated Sine-Generated Distributions

### 2.1. Presentation

The exponentiation technique [14] is one of the popular techniques that received extensive consideration in extending classical distributions for better flexibility. In this work, we apply the exponentiation technique to the SG family. To begin, the SG family, introduced by [7], has the following CDF:

$$H\left(x\right)=H\left(x;\alpha ,\xi \right)=\sin\left(\frac{\pi }{2}G\left(x;\xi \right)\right),\;x\in R,$$

where G(x;ξ) is any valid CDF of a continuous distribution, and ξ is a vector of parameters. Therefore, we propose the exponentiated sine-G (ESG) distribution with CDF given by

(1) $$\begin{array}{c} F\left(x\right)=F\left(x;\alpha ,\xi \right)={\left[\sin\left(\frac{\pi }{2}G\left(x;\xi \right)\right)\right]}^{\alpha },\;\alpha >0. \end{array}$$

Notice that, if α=1, the ESG family is reduced to the SG family.

The probability density function (PDF) connected to the SG family is

(2) $$\begin{array}{c} f\left(x\right)=f\left(x;\alpha ,\xi \right)=\frac{\alpha \pi }{2}g\left(x;\xi \right)\cos\left(\frac{\pi }{2}G\left(x;\xi \right)\right){\left[\sin\left(\frac{\pi }{2}G\left(x;\xi \right)\right)\right]}^{\alpha -1}, \end{array}$$

where g(x;ξ) is the PDF of G(x;ξ).

The related survival function (SF) and hazard rate function (HRF) are derived as

$$\begin{array}{cc} s(x) & =s\left(x;\alpha ,\xi \right)=1-{\left[\sin\left(\frac{\pi }{2}G\left(x;\xi \right)\right)\right]}^{\alpha } \end{array}$$

and

$$\begin{array}{cc} h(x) & =h\left(x;\alpha ,\xi \right)=\frac{\left(\alpha \pi /2\right)g\left(x;\xi \right)\cos\left((\pi /2)G(x;\xi )\right){\left[\sin\left((\pi /2)G(x;\xi )\right)\right]}^{\alpha -1}}{1-{\left[\sin\left((\pi /2)G(x;\xi )\right)\right]}^{\alpha }}, \end{array}$$

respectively.

As a first approach on the impact of the parameter α, we can notice that, for α≥1, F(x)≤F(x;1,ξ). The contrary holds for 0<α<1. In addition, for a sufficiently small *x*, i.e., as G(x;ξ)→0, $F\left(x\right)∼{\left(\pi /2\right)}^{\alpha }G^{\alpha }\left(x;\xi \right)$ and $f\left(x\right)∼\alpha {\left(\pi /2\right)}^{\alpha }g\left(x;\xi \right)G^{\alpha -1}\left(x;\xi \right)$. On the other hand, for a sufficiently large *x*, i.e., as G(x;ξ)→1, then $s\left(x\right)∼\left(\alpha \pi^{2}/8\right){\left(1-G\left(x;\xi \right)\right)}^{2}$, and $f\left(x\right)∼\left(\alpha \pi^{2}/4\right)g\left(x;\xi \right)\left(1-G\left(x;\xi \right)\right)$.

Let us now perform a basic quantile analysis of the ESG family. Firstly, we can use the quantile function (QF) of the distribution to analyze its skewness and kurtosis; it also serves as a means of parameter estimation. Moreover, it is used for generating random data that follows a specified distribution. The QF aided in the computations of some distribution properties and goodness-of-fit measures. After some developments, the QF of the ESG family is given by

$$\begin{array}{c} Q\left(p\right)=Q\left(p;\xi \right)=G^{-1}\left(\left[\frac{2}{\pi }\arcsin\left(p^{1/\alpha }\right)\right];\xi \right),\;0<p<1, \end{array}$$

where $G^{-1}\left(x;\xi \right)$ is the QF related to G(x;ξ). In particular, the median is

$$Q_{*}=Q\left(\frac{1}{2}\right)=G^{-1}\left(\left[\frac{2}{\pi }\arcsin\left(2^{-1/\alpha }\right)\right];\xi \right).$$

Obviously, the definition of G(x;ξ) is crucial to the modeling capabilities of the ESG family. A precise example will be presented later.

### 2.2. Useful Expansion

In this part, we present the PDF of the ESG family in a series expansion form. To achieve this aim, some preliminary results are required. First, we recall that the sine and cosine series expansions are given by $\sin x=\sum_{n=0}^{\infty }{\left(-1\right)}^{n}x^{2n+1}/(2n+1)!$ and $\cos x=\sum_{n=0}^{\infty }{\left(-1\right)}^{n}x^{2n}/(2n)!$. We will also need the power series raised to the power of integer as given by [15]. It is recalled in the following lemma.

**Lemma** **1** ([15]). *For a given power series of the form $\sum_{k=0}^{\infty }a_{k}x^{k}$, where n denotes a positive integer, we have*

(3) $$\begin{array}{c} {\left(\sum_{k=0}^{\infty }a_{k}x^{k}\right)}^{n}=\sum_{k=0}^{\infty }c_{k}x^{k}, \end{array}$$

*where $c_{0}=a_{0}^{n}$, $c_{m}=\left[1/\left(ma_{0}\right)\right]\sum_{k=1}^{m}\left(kn-m+k\right)a_{k}c_{m-k}$ for m≥1.*

The following lemma is for future technical purposes.

**Lemma** **2.** *We have*

(4) $$\begin{array}{c} A_{n}=\sum_{n=1}^{\infty }\frac{{\left(-1\right)}^{n}{\left(\pi /2\right)}^{2n}}{(2n+1)!}=\frac{2}{\pi }-1\approx -0.36338. \end{array}$$

**Proof.**  We can prove that $A_{n}$ is a convergent alternating series via standard convergence criteria, and the value of $A_{n}$ can be verified from https://www.wolframalpha.com/widgets/gallery/view.jsp?id=d983db47634e1936eb568b79884012ac (accessed on 18 August 2021) or https://www.emathhelp.net/calculators/calculus-2/series-calculator/ (accessed on 18 August 2021). □

A series expansion of the exponentiated version of a sine term is provided below.

**Lemma** **3.** *Let α>0 be real and noninteger, and x such that 0<G(x;ξ)<1, then*

sinπ2G(x;ξ)α−1=π2α−1Gα−1(x;ξ)+∑l=1∞∑i=0∞α−1lπ2α−1biG2(i+l)+α−1(x;ξ),

*where $b_{0}=a_{0}^{l}$, $b_{m}=\left[1/\left(ma_{0}\right)\right]\sum_{i=1}^{m}\left(il-m+i\right)a_{i}b_{m-i}$ for m≥1, and $a_{i}={\left(-1\right)}^{i+1}{\left(\pi /2\right)}^{2i+2}$ /(2i+3)!.*

**Proof.**  We can write the sine series expansion by separating the first term as

$$\begin{array}{cc} {\left[\sin\left(\frac{\pi }{2}G\left(x;\xi \right)\right)\right]}^{\alpha -1} & ={\left[\frac{\pi }{2}G\left(x;\xi \right)+\sum_{n=1}^{\infty }\frac{{\left(-1\right)}^{n}{\left(\frac{\pi }{2}\right)}^{2n+1}}{(2n+1)!}G^{2n+1}\left(x;\xi \right)\right]}^{\alpha -1}\\ \; & ={\left(\frac{\pi }{2}\right)}^{\alpha -1}G^{\alpha -1}\left(x;\xi \right){\left[1+\sum_{n=1}^{\infty }\frac{{\left(-1\right)}^{n}{\left(\frac{\pi }{2}\right)}^{2n}}{(2n+1)!}G^{2n}\left(x;\xi \right)\right]}^{\alpha -1}. \end{array}$$

By considering Equation (4), we can conclude that

$\left|\sum_{n=1}^{\infty }\left[{\left(-1\right)}^{n}{\left(\pi /2\right)}^{2n}/(2n+1)!\right]G^{2n}\left(x;\xi \right)\right|<1$, since $0<G^{2n}\left(x;\xi \right)<1$ for all *x*. Let n=i+1, and i≥0, then, n≥1. Therefore,

$$\begin{array}{c} {\left[\sin\left(\frac{\pi }{2}G\left(x;\xi \right)\right)\right]}^{\alpha -1}={\left(\frac{\pi }{2}\right)}^{\alpha -1}G^{\alpha -1}\left(x;\xi \right){\left[1+\sum_{i=0}^{\infty }a_{i}G^{2i+2}\left(x;\xi \right)\right]}^{\alpha -1}, \end{array}$$

where $a_{i}={\left(-1\right)}^{i+1}{\left(\pi /2\right)}^{2i+2}/(2i+3)!$. By the generalized binomial expansion and some algebra, we get

sinπ2G(x;ξ)α−1=π2α−1Gα−1(x;ξ)1+∑l=1∞α−1lG2l(x;ξ)∑i=0∞aiG2i(x;ξ)l.

Since l≥1 is an integer, we can apply Equation (3) to the series with the power *l*, thus,

sinπ2G(x;ξ)α−1=π2α−1Gα−1(x;ξ)1+∑l=1∞∑i=0∞α−1lbiG2(i+l)(x;ξ),

where $b_{0}=a_{0}^{l}$ and $b_{m}=\left[1/\left(ma_{0}\right)\right]\sum_{i=1}^{m}\left(il-m+i\right)a_{i}b_{m-i}$ for m≥1. Hence,

sinπ2G(x;ξ)α−1=π2α−1Gα−1(x;ξ)+∑l=1∞∑i=0∞α−1lπ2α−1biG2(i+l)+α−1(x;ξ).

The stated result is obtained.  □

Now, we can apply Lemma 3 and the series expansion $\cos\left((\pi /2)G(x;\xi )\right)=\sum_{k=0}^{\infty }$$\left[{\left(-1\right)}^{k}{\left(\pi /2\right)}^{2k}/(2k)!\right]G^{2k}\left(x;\xi \right)$ to represent the PDF in Equation (2) as

(5) $$\begin{array}{c} f\left(x\right)=\sum_{k=0}^{\infty }D_{k}^{\left(1\right)}g\left(x;\xi \right)G^{2k+\alpha -1}\left(x;\xi \right)+\sum_{k,i=0}^{\infty }\sum_{l=1}^{\infty }D_{i,k,l}^{\left(2\right)}g\left(x;\xi \right)G^{2(i+l+k)+\alpha -1}\left(x;\xi \right), \end{array}$$

where $D_{k}^{\left(1\right)}=\alpha {\left(-1\right)}^{k}{\left(\pi /2\right)}^{2k+\alpha }/(2k)!$, Di,k,l(2)=α(−1)k(π/2)2k+αα−1lbi/(2k)! and $b_{i}$ is given in Lemma 3. Most of the important properties of the ESG family can be represented in a closed form analytically. The PDF is now a series of PDFs of the exponentiated baseline distribution.

### 2.3. Moments and Entropy

In this subsection, moments and Rényi entropy of the ESG family are derived. Let *X* be a random variable (RV) with distribution belonging to the ESG family. Then, the *r*th moment of *X* is traditionally obtained in the following way: $\mu_{r}=E\left(X^{r}\right)=\int_{-\infty }^{\infty }x^{r}f\left(x\right)dx$. By virtue of Equation (5), the following series expansion holds:

(6) $$\begin{array}{cc} \mu_{r} & =\sum_{k=0}^{\infty }D_{k}^{\left(1\right)}\int_{-\infty }^{\infty }x^{r}g\left(x;\xi \right)G^{2k+\alpha -1}\left(x;\xi \right)dx\\ \; & +\sum_{k,i=0}^{\infty }\sum_{l=1}^{\infty }D_{i,k,l}^{\left(2\right)}\int_{-\infty }^{\infty }x^{r}g\left(x;\xi \right)G^{2(i+l+k)+\alpha -1}\left(x;\xi \right)dx. \end{array}$$

Specific moments can be determined by setting r=1,2,3,….

Entropy, on the other hand, is a measure of a system’s disorder or randomness. The Rényi entropy of *X* is defined by

$$I_{R(\rho )}=\frac{1}{1-\rho }\log\left[\int_{-\infty }^{\infty }f^{\rho }\left(x\right)dx\right],\;\rho >0,\rho \neq 1.$$

Thus, to determine $I_{R(\rho )}$, it is enough to compute $\int_{-\infty }^{\infty }f^{\rho }\left(x\right)dx$, and to plug it into the previous logarithmic-integral expression. To begin, we have

(7) $$\begin{array}{c} f^{\rho }\left(x\right)={\left(\frac{\alpha \pi }{2}\right)}^{\rho }g^{\rho }\left(x;\xi \right)\cos^{\rho }\left(\frac{\pi }{2}G\left(x;\xi \right)\right)\sin^{\rho (\alpha -1)}\left(\frac{\pi }{2}G\left(x;\xi \right)\right). \end{array}$$

For an expansion of the exponentiated cosine term, the following lemma is useful.

**Lemma** **4.** *Let ρ>0 real and noninteger. Then, we have*

(8) $$\begin{array}{cc} \cos^{\rho }\left(\frac{\pi }{2}G\left(x;\xi \right)\right) & =1+\sum_{j=1}^{\infty }\sum_{k=0}^{\infty }z_{k,j}^{**}G^{2(k+j)}\left(x;\xi \right), \end{array}$$

where zk,j**=ρjzk*, $z_{0}^{*}=z_{0}^{j}$, $z_{m}^{*}=\left[1/\left(mz_{0}\right)\right]\sum_{k=1}^{m}\left(kj-m+k\right)z_{k}z_{m-k}^{*}$ for m≥1, and $z_{k}={\left(-1\right)}^{k+1}{\left(\pi /2\right)}^{2(k+1)}/\left(2\left(k+1\right)\right)!.$

**Proof.**  Applying some algebra to the cosine series expansion with n≥0 and replacing n=k+1, k≥0, then n≥1, as in previous lemmas, we have

cosρπ2G(x;ξ)=∑j=0∞ρjcosπ2G(x;ξ)−1j=1+∑j=1∞ρj∑n=0∞(−1)n(π/2)2n(2n)!G2n(x;ξ)−1j=1+∑j=1∞ρj∑k=0∞(−1)k+1(π/2)2(k+1)(2(k+1))!G2(k+1)(x;ξ)j=1+∑j=1∞ρjG2j(x;ξ)∑k=0∞zkG2k(x;ξ)j,

where $z_{k}={\left(-1\right)}^{k+1}{\left(\pi /2\right)}^{2(k+1)}/\left(2\left(k+1\right)\right)!$. Since j≥1, we can use Equation (3), which yields

cosρπ2G(x;ξ)=1+∑j=1∞ρj∑k=0∞zk*G2(k+j)(x;ξ),

where $z_{0}^{*}=z_{0}^{j}$ and $z_{m}^{*}=\left[1/\left(mz_{0}\right)\right]\sum_{k=1}^{m}\left(kj-m+k\right)z_{k}z_{m-k}^{*}$ for m≥1. Hence,

$$\begin{array}{cc} \cos^{\rho }\left(\frac{\pi }{2}G\left(x;\xi \right)\right) & =1+\sum_{j=1}^{\infty }\sum_{k=0}^{\infty }z_{k,j}^{**}G^{2(k+j)}\left(x;\xi \right), \end{array}$$

where zk,j**=ρjzk*. The stated expansion is proved.  □

On the other hand, with a slight adaptation of Lemma 3, we have

(9) $$\begin{array}{cc} {\left[\sin\left(\frac{\pi }{2}G\left(x;\xi \right)\right)\right]}^{\rho (\alpha -1)} & ={\left(\frac{\pi }{2}\right)}^{\rho (\alpha -1)}G^{\rho (\alpha -1)}\left(x;\xi \right)+\sum_{l=1}^{\infty }\sum_{i=0}^{\infty }V_{i,l}G^{2(i+l)+\rho (\alpha -1)}\left(x;\xi \right), \end{array}$$

where Vi,l=ρ(α−1)l(π/2)ρ(α−1)bi and $b_{i}$ is similar to that of Lemma 3. Thus, by substituting Lemma 4 and Equation (9) in Equation (7), we can compute the integral of $f^{\rho }\left(x\right)$ to get the Rényi entropy.

The Shannon entropy can be expressed in a similar series expansion manner.

## 3. Stress-Strength Reliability

The system performance parameter, known as the stress-strength parameter, is crucial in the context of a system’s mechanical reliability. In practice, a good design ensures that the strength exceeds the anticipated stress. If a component has stress modeled by an RV *X* and is subjected to strength modeled by an RV *Y*, the stress-strength parameter defined by R=P(Y<X) determines the system performance. The system will fail if and only if the applied stress exceeds the system’s strength. On the other hand, *R* can be used to compare two populations. Engineering, biological sciences, and finance all have uses for stress-strength reliability parameters. See [16] (Chapter 7), among others.

The parameter *R* was studied by many authors in literature through various viewpoints. Assuming that *X* and *Y* are independent, the following distributions have been considered: generalized logistic distribution (see [17]), normal distribution (see [18,19]), exponential distribution with the common location parameter (see [20]), generalized exponential distribution (see [21]), generalized exponential Poisson distribution (see [22]), Poisson-odd generalized exponential distribution (see [23]), beta-Erlang truncated exponential distribution (see [24]), Poisson-generalized half logistic distribution (see [25]), Poisson half logistic distribution (see [26]), and Weibull (W) distribution (see [27,28,29]), among others.

Here, we study the expression of *R* in the setting of the ESG family. Suppose that *X* has the PDF $f(x;\alpha_{1},\xi )$, *Y* has the CDF $F(y;\alpha_{2},\xi )$, and *X* and *Y* are assumed to be independent. Then, the stress-strength reliability parameter is then defined as follows:

$$R=P\left(Y<X\right)=\int_{-\infty }^{\infty }f\left(x;\alpha_{1},\xi \right)F\left(x;\alpha_{2},\xi \right)dx.$$

Thus,

(10) $$\begin{array}{cc} R & =\alpha_{1}\int_{-\infty }^{\infty }\frac{\pi }{2}g\left(x;\xi \right)\cos\left(\frac{\pi }{2}G\left(x;\xi \right)\right){\left[\sin\left(\frac{\pi }{2}G\left(x;\xi \right)\right)\right]}^{\alpha_{1}+\alpha_{1}-1}dx=\frac{\alpha_{1}}{\alpha_{1}+\alpha_{2}}. \end{array}$$

We see that *R* has some manageable form that can make it easy for statistical purposes, and so on. In one of the next sections, we discuss the behavior of *R* with some particular distribution of the ESG family for illustration.

## 4. The ES Weibull Distribution

In this section, we concentrate our attention on one special distribution of the ESG family, called the ESW distribution or ESW(α,β,λ) distribution. In this case, the baseline in Equation (1) is the W distribution with CDF and PDF given as

$$\begin{array}{cc} G(x;\beta ,\lambda ) & =1-e^{-\lambda x^{\beta }},\;\beta ,\lambda ,x>0, \end{array}$$

and

$$\begin{array}{cc} g(x;\beta ,\lambda ) & =\beta \lambda x^{\beta -1}e^{-\lambda x^{\beta }},\;x>0, \end{array}$$

respectively. Therefore, the CDF of the ESW distribution has the following expression:

(11) $$\begin{array}{c} F\left(x\right)=F\left(x;\alpha ,\beta ,\lambda \right)={\left[\sin\left(\frac{\pi }{2}\left(1-e^{-\lambda x^{\beta }}\right)\right)\right]}^{\alpha },\;\alpha ,\beta ,\lambda ,x>0. \end{array}$$

**Figure 1:**
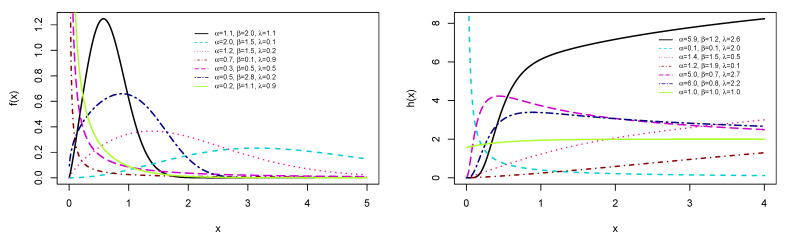
Selected plots of PDF f(x) and HRF h(x).

**Figure 2:**
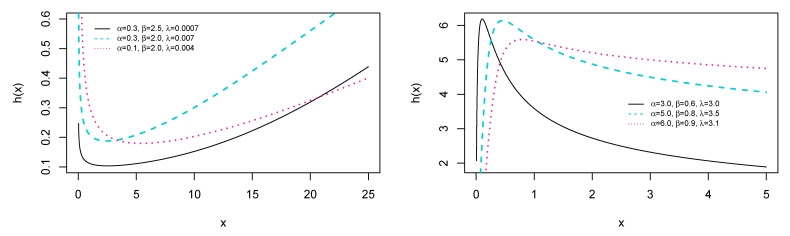
Selected plots of various kinds of bathtub shapes of HRF h(x).

If β=1, the ESW distribution is reduced to the ES exponential (ESE) distribution. The corresponding probability density, survival, and hazard rate functions are expressed by

(12) $$\begin{array}{cc} f(x) & =f\left(x;\alpha ,\beta ,\lambda \right)=\frac{\pi }{2}\alpha \beta \lambda x^{\beta -1}e^{-\lambda x^{\beta }}\cos\left(\frac{\pi }{2}\left(1-e^{-\lambda x^{\beta }}\right)\right){\left[\sin\left(\frac{\pi }{2}\left(1-e^{-\lambda x^{\beta }}\right)\right)\right]}^{\alpha -1}, \end{array}$$

(13) $$\begin{array}{cc} s(x) & =s\left(x;\alpha ,\beta ,\lambda \right)=1-{\left[\sin\left(\frac{\pi }{2}\left(1-e^{-\lambda x^{\beta }}\right)\right)\right]}^{\alpha } \end{array}$$

and

(14) $$\begin{array}{cc} h(x) & =h(x;\alpha ,\beta ,\lambda )\\ \; & =\frac{\left(\pi /2\right)\alpha \beta \lambda x^{\beta -1}e^{-\lambda x^{\beta }}\cos\left(\left(\pi /2\right)\left(1-e^{-\lambda x^{\beta }}\right)\right){\left[\sin\left(\left(\pi /2\right)\left(1-e^{-\lambda x^{\beta }}\right)\right)\right]}^{\alpha -1}}{1-{\left[\sin\left(\left(\pi /2\right)\left(1-e^{-\lambda x^{\beta }}\right)\right)\right]}^{\alpha }}, \end{array}$$

respectively. Figure 1 and Figure 2 show the plot of the PDF given by Equation (12) and HRF specified in Equation (14). In particular, Figure 2 shows that the failure rate is flexible enough to accommodate monotonic (increasing and decreasing) shapes, and also bathtub and upside-down bathtub shapes.

The decreasing behavior of the PDF is formulated in the following result.

**Theorem** **1.** *The PDF of the ESW distribution given by Equation (12) is a decreasing function for α≤1 and β≤1.*

**Proof.**  We have

$$\begin{array}{cc} {\left\{\log\left[f\left(x\right)\right]\right\}}^{\prime } & =\frac{\beta -1}{x}-\beta \lambda x^{\beta -1}-\frac{\left(\pi /2\right)\alpha \beta \lambda x^{\beta -1}e^{-\lambda x^{\beta }}\sin\left(\left(\pi /2\right)\left(1-e^{-\lambda x^{\beta }}\right)\right)}{\cos\left(\left(\pi /2\right)\left(1-e^{-\lambda x^{\beta }}\right)\right)}\\ \; & +\frac{\left(\alpha -1\right)\left(\pi /2\right)\alpha \beta \lambda x^{\beta -1}e^{-\lambda x^{\beta }}\cos\left(\left(\pi /2\right)\left(1-e^{-\lambda x^{\beta }}\right)\right)}{\sin\left(\left(\pi /2\right)\left(1-e^{-\lambda x^{\beta }}\right)\right)}. \end{array}$$

When α≤1 and β≤1, all the terms in the sum are negative, implying that ${\left\{\log\left[f\left(x\right)\right]\right\}}^{\prime }\leq 0$, so log[f(x)] is decreasing, and the same for f(x). This ends the proof.  □

**Proposition** **1.** *The asymptotic behavior of F(x) in Equation (11), f(x) in Equation (12) and s(x) in Equation (13) are as follows:*

- *As x→0, we have*
(15) $$\begin{array}{c} F\left(x\right)∼{\left(\frac{\pi }{2}\right)}^{\alpha }G^{\alpha }\left(x;\beta ,\lambda \right)∼{\left(\frac{\pi }{2}\right)}^{\alpha }\lambda^{\alpha }x^{\alpha \beta } \end{array}$$
  *and*
$$\begin{array}{c} f\left(x\right)∼\alpha {\left(\frac{\pi }{2}\right)}^{\alpha }g\left(x;\beta ,\lambda \right)G^{\alpha -1}\left(x;\beta ,\lambda \right)∼{\left(\frac{\pi }{2}\right)}^{\alpha }\lambda^{\alpha }\alpha \beta x^{\alpha \beta -1}. \end{array}$$
- *As x→∞, we have*
(16) $$\begin{array}{c} s\left(x\right)∼\frac{\alpha \pi^{2}}{8}{\left(1-G\left(x;\beta ,\lambda \right)\right)}^{2}=\frac{\alpha \pi^{2}}{8}e^{-2\lambda x^{\beta }} \end{array}$$
  *and*
$$\begin{array}{c} f\left(x\right)∼\frac{\alpha \pi^{2}}{4}g\left(x;\beta ,\lambda \right)\left(1-G\left(x;\beta ,\lambda \right)\right)=\frac{\alpha \pi^{2}}{4}\lambda \beta x^{\beta -1}e^{-2\lambda x^{\beta }}. \end{array}$$

**Proof.**  Standard equivalency function findings are used in the proof. For this reason, the details are omitted.  □

### 4.1. Quantile and Moments

The QF of the ESW distribution can be derived from Equation (11) as

(17) $$\begin{array}{c} Q\left(p\right)=Q\left(p;\beta ,\lambda \right)={\left[-\frac{1}{\lambda }\log\left(1-\frac{2}{\pi }\arcsin\left(p^{1/\alpha }\right)\right)\right]}^{1/\beta },\;0<p<1. \end{array}$$

The median of the ESW distribution is

$$Q_{*}=Q\left(\frac{1}{2}\right)={\left[-\frac{1}{\lambda }\log\left(1-\frac{2}{\pi }\arcsin\left(2^{-1/\alpha }\right)\right)\right]}^{1/\beta }.$$

**Figure 3:**
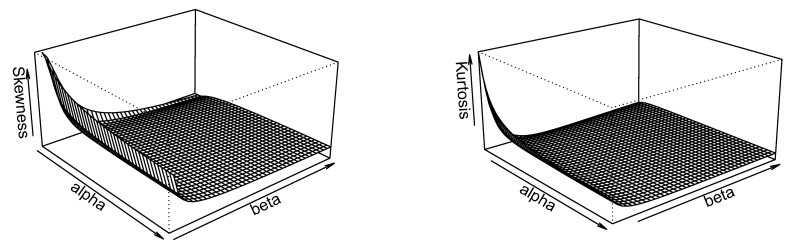
Selected plots of B and M of the ESW distribution.

The skewness and kurtosis properties of the ESW distribution can be determined using the Bowley’s skewness and Moor’s kurtosis, among other quantile based measures. They are defined by

$$\begin{array}{c} B=\frac{Q(3/4)-2Q(2/4)+Q(1/4)}{Q(3/4)-Q(1/4)},\;M=\frac{Q(7/8)-Q(5/8)+Q(3/8)-Q(1/8)}{Q(6/8)-Q(2/8)}, \end{array}$$

respectively. Equation (17) shows that both B and M are independent of λ. Figure 3 reveals that these quantile skewness and kurtosis are decreasing with respect to α and β.

The moments of the ESW distribution can be computed from $\mu_{r}=\int_{-\infty }^{\infty }x^{r}f\left(x\right)dx$. By letting $u=\sin\left(\left(\pi /2\right)\left(1-e^{-\lambda x^{\beta }}\right)\right)$, this integral is reduced to

(18) $$\begin{array}{c} \mu_{r}=\alpha \lambda^{-r/\beta }\int_{0}^{1}{\left[-\log\left(1-\frac{2}{\pi }\arcsin\left(u\right)\right)\right]}^{r/\beta }u^{\alpha -1}du. \end{array}$$

It is not known if the integral in Equation (18) can be derived analytically, but it can be computed numerically by software such as R. Further, to obtain the moments of the ESW distribution in a series form, we can use Equation (6), and the following lemma.

**Lemma** **5.** *Let $\omega_{1}\in R$, $\omega_{2}>0$, $\omega_{3}>0$ real and noninteger. We define*

$$\begin{array}{c} \psi \left(\omega_{1},\omega_{2},\omega_{3}\right)=\int_{0}^{\infty }x^{\omega_{1}}g^{\omega_{2}}\left(x;\beta ,\lambda \right)G^{\omega_{3}}\left(x;\beta ,\lambda \right)dx. \end{array}$$

*Then,*

(19) ψ(ω1,ω2,ω3)=∑s=0∞λω2βω2−1ω3s(−1)s[λ(ω2+s)](1+ω1−ω2)/β+ω2Γ1β(1+ω1−ω2)+ω2,

*where Γ(x) refers to the standard gamma function.*

**Proof.**  In an expanded form, we have

ψ(ω1,ω2,ω3)=λω2βω2∫0∞xω2(β−1)+ω1e−λω2xβ(1−e−λxβ)ω3dx=λω2βω2∑s=0∞ω3s(−1)s∫0∞xω2(β−1)+ω1e−λ(ω2+s)xβdx.

By setting $u=\lambda \left(\omega_{2}+s\right)x^{\beta }$, we get

ψ(ω1,ω2,ω3)=∑s=0∞λω2βω2−1ω3s(−1)s[λ(ω2+s)](1+ω1−ω2)/β+ω2∫0∞u(1+ω1−ω2)/β+ω2−1e−udu=∑s=0∞λω2βω2−1ω3s(−1)s[λ(ω2+s)](1+ω1−ω2)/β+ω2Γ1β(1+ω1−ω2)+ω2.

The stated result is obtained.  □

In fact, most of the features of the ESW distribution may be obtained in a series form using this lemma. In particular, Equations (5) and (19) can be used to express the moments of the ESW distribution in a series of the following form:

$$\begin{array}{cc} \mu_{r} & =\sum_{k=0}^{\infty }D_{k}^{\left(1\right)}\psi \left(r,1,2k+\alpha -1\right)+\sum_{k,i=0}^{\infty }\sum_{l=1}^{\infty }D_{i,k,l}^{\left(2\right)}\psi \left(r,1,2(i+l+k)+\alpha -1\right), \end{array}$$

where $D_{k}^{\left(1\right)}$ and $D_{i,k,l}^{\left(2\right)}$ are given in Equation (5) and $\psi (\omega_{1},\omega_{2},\omega_{3})$ follows from Lemma 5.

### 4.2. Moments of Residual Life

Residual life functions are involved in a variety of fields, including engineering, quality control, and life testing. They also aid in the determination of the asymptotic distributions of order statistics. For an RV *X* with the ESW distribution, the related mean residual life is defined by $\zeta \left(t\right)=E\left(X-t|X>t\right)=\int_{0}^{\infty }s(x+t)/s(t)dx$. The reversed mean residual life of *X* is defined by $\bar{\zeta }\left(t\right)=E\left(t-X|X\leq t\right)=\int_{0}^{t}F(x)/F(t)dx$. Now, we derive some asymptotic properties of ζ(t) for sufficiently large *t*, and asymptotic properties of $\bar{\zeta }\left(t\right)$ for very small *t*.

**Theorem** **2.** *Let X be an RV with the ESW distribution. Then, for t→∞, the mean residual life of X satisfies*

$$\begin{array}{c} \zeta \left(t\right)∼\frac{e^{2\lambda t^{\beta }}}{{\left(2\lambda \right)}^{1/\beta }\beta }\Gamma \left(\frac{1}{\beta },2\lambda t^{\beta }\right), \end{array}$$

*where Γ(a,b) denotes the incomplete (upper version) gamma function.*

**Proof.**  From the asymptotic behavior of s(t) for t→∞ in Equation (16), we have

$$\begin{array}{c} \zeta \left(t\right)=\int_{0}^{\infty }\frac{s(x+t)}{s(t)}dx∼e^{2\lambda t^{\beta }}\int_{0}^{\infty }e^{-2\lambda {\left(x+t\right)}^{\beta }}dx. \end{array}$$

By applying the change of variable $u=2\lambda {\left(x+t\right)}^{\beta }$, and after some algebra, we get

$$\begin{array}{c} \zeta \left(t\right)∼\frac{e^{2\lambda t^{\beta }}}{{\left(2\lambda \right)}^{1/\beta }\beta }\int_{2\lambda t^{\beta }}^{\infty }u^{1/\beta -1}e^{-u}du=\frac{e^{2\lambda t^{\beta }}}{{\left(2\lambda \right)}^{1/\beta }\beta }\Gamma \left(\frac{1}{\beta },2\lambda t^{\beta }\right). \end{array}$$

The stated equivalence is obtained.  □

**Theorem** **3.** *Let X be an RV with the ESW distribution. Then, for t→0, the reversed mean residual life of X satisfies*

$$\begin{array}{c} \lim_{t\to 0}\bar{\zeta }\left(t\right)∼\frac{t}{\alpha \beta +1}. \end{array}$$

**Proof.**  From the asymptotic behavior of F(t) for t→0 in Equation (15), we have

$$\bar{\zeta }\left(t\right)=\int_{0}^{t}\frac{F(x)}{F(t)}dx∼\int_{0}^{t}\frac{x^{\alpha \beta }}{t^{\alpha \beta }}dx=\frac{t}{\alpha \beta +1}.$$

The desired outcome is demonstrated.  □

### 4.3. Order Statistics and Asymptotic

We now focus on the order statistics from the ESW distribution, and some of their asymptotic properties. Let $X_{1},X_{2},\ldots ,X_{n}$, n≥1, be a sample of independent RVs following the ESW distribution. Let us consider $X_{1:n},X_{2:n},\ldots ,X_{n:n}$ the ordered versions of $X_{1},X_{2},\ldots ,X_{n}$. Then, for any j=1,2,…,n, the PDF of $X_{j:n}$ is given as

(20) $$\begin{array}{cc} f_{j:n}\left(x\right) & =\frac{n!}{(j-1)!(n-j)!}f(x)F^{j-1}\left(x\right)s^{n-j}\left(x\right),\\ \; & =\sum_{m=0}^{n-j}\frac{n!{\left(-1\right)}^{m}}{(j-1)!(n-j-m)!m!}f(x)F^{j+m-1}\left(x\right). \end{array}$$

Therefore, by expressing F(x) as in Equation (11), and f(x) as in Equation (12) into Equation (20), we have

$$\begin{array}{c} f_{j:n}\left(x\right)=\sum_{m=0}^{n-j}\frac{{\left(-1\right)}^{m}\alpha \beta \lambda \pi n!x^{\beta -1}e^{-\lambda x^{\beta }}}{2(j-1)!(n-j-m)!m!}\cos\left(\frac{\pi }{2}\left(1-e^{-\lambda x^{\beta }}\right)\right){\left[\sin\left(\frac{\pi }{2}\left(1-e^{-\lambda x^{\beta }}\right)\right)\right]}^{\alpha (j+m)-1}. \end{array}$$

Also, we can express $f_{j:n}\left(x\right)$ as a series expansion depending on PDFs of the ESW distribution with parameters α(j+m), β and λ as

$$\begin{array}{c} f_{j:n}\left(x\right)=\sum_{m=0}^{n-j}\frac{n!{\left(-1\right)}^{m}}{(j+m)(j-1)!(n-j-m)!m!}f\left(x;\alpha \left(j+m\right),\beta ,\lambda \right). \end{array}$$

The asymptotic distributions for the minimum order statistic $\left(X_{1:n}\right)$ can be derived as follows. For detail see [30] (Chapter 8).

**Theorem** **4.** Let $X_{1},X_{2},\ldots ,X_{n}$, n≥1, be a sample of independent RVs following the ESW distribution. Let $X_{1:n}=\inf\left(X_{1},\ldots ,X_{n}\right)$ and $B_{n}^{*}=\left(X_{1:n}-a_{n}^{*}\right)/b_{n}^{*}$. Then, for any x>0, we have

$$\begin{array}{c} \lim_{n\to \infty }P\left(B_{n}^{*}\leq x\right)=1-e^{-x^{\alpha \beta }}. \end{array}$$

*We recognize the CDF of the W distribution with parameters* 1 and αβ. The normalizing constant can be derived from Equation (17) by following Theorem 8.3.6 of [30]. That is, we take $a_{n}^{*}=0$ and $b_{n}^{*}=Q\left(1/n\right)$.

**Proof.**  We aim to apply Theorem 8.3.6 of [30]. By Equation (15), we have

$$\begin{array}{c} \lim_{\epsilon \to 0}\frac{F(\epsilon x)}{F(\epsilon )}∼\lim_{\epsilon \to 0}\frac{{\left(\epsilon x\right)}^{\alpha \beta }}{\epsilon^{\alpha \beta }}=x^{\alpha \beta }. \end{array}$$

It follows from Theorem 8.3.6 of [30] the desired result. This ends the proof.  □

## 5. Parameter Estimation

In this part, we use maximum likelihood and Bayes estimation approaches to estimate the new model parameters. The maximum likelihood estimation of the ESG family is discussed in the general case, while the Bayes estimation is discussed for the ESW distribution only. The estimation techniques are examined by simulation studies.

The expression of the reliability parameter *R* of the ESW distribution is derived under a common scale parameter from two independent RVs with possibly different ESW distributions. Subsequently, the maximum likelihood estimation of *R* is discussed, and the nonparametric bootstrap confidence interval (CI) is used to determine the approximate CI of *R*, and finally assessed by simulation studies.

### 5.1. Maximum Likelihood Estimation

The method of maximum likelihood can be used to estimate the parameters of the models coming from the ESG family. To begin, let $X_{1},X_{2},\ldots ,X_{n}$, n≥1, be a sample of independent RVs whose distribution belongs to the ESG family. We denote by $x_{1},x_{2},\ldots ,x_{n}$ the corresponding observations. Let $\theta ={\left(\alpha ,\xi \right)}^{T}$ be a vector of parameters. Then, the maximum likelihood estimate (MLE) vector of θ, say $\hat{\theta }={\left(\hat{\alpha },\hat{\xi }\right)}^{T}$, is obtained by the maximization of the log-likelihood function given by

$$\begin{array}{cc} L(\theta ) & =n\log\alpha +n\log\left(\frac{\pi }{2}\right)+\sum_{i=1}^{n}\log g\left(x_{i};\xi \right)+\sum_{i=1}^{n}\log\left[\cos\left(\frac{\pi }{2}G\left(x_{i};\xi \right)\right)\right]\\ \; & +\left(\alpha -1\right)\sum_{i=1}^{n}\log\left[\sin\left(\frac{\pi }{2}G\left(x_{i};\xi \right)\right)\right], \end{array}$$

with respect to θ. This maximization can be achieved by solving the following nonlinear system of equations given as

(21) $$\begin{array}{cc} \frac{\partial L(\theta )}{\partial \alpha } & =\frac{n}{\alpha }+\sum_{i=1}^{n}\log\left[\sin\left(\frac{\pi }{2}G\left(x_{i};\xi \right)\right)\right]=0, \end{array}$$

and   

$$\begin{array}{cc} \frac{\partial L(\theta )}{\partial \xi } & =\sum_{i=1}^{n}\frac{g^{\xi^{\prime }}\left(x_{i};\xi \right)}{g(x_{i};\xi )}-\frac{\pi }{2}\sum_{i=1}^{n}G^{\xi^{\prime }}\left(x_{i};\xi \right)\tan\left(\frac{\pi }{2}G\left(x_{i};\xi \right)\right)\\ \; & +\frac{\pi }{2}\left(\alpha -1\right)\sum_{i=1}^{n}G^{\xi^{\prime }}\left(x_{i};\xi \right)\cot\left(\frac{\pi }{2}G\left(x_{i};\xi \right)\right)=0, \end{array}$$

where $g^{\xi^{\prime }}\left(x_{i};\xi \right)=\partial g\left(x_{i};\xi \right)/\partial \xi$ and $G^{\xi^{\prime }}\left(x_{i};\xi \right)=\partial G\left(x_{i};\xi \right)/\partial \xi$. Now, we can analyze the existence and uniqueness of the MLE of the parameter α. For more studies on the existence and uniqueness of the MLEs for various models, one can see [25,31,32], among others.

**Proposition** **2.** *Let D(α;ξ) represent the right-hand side of Equation (21), and suppose that ξ are true values of the parameters. Then, D(α;ξ)=0 has a unique real root.*

**Proof.**  Equation (21) shows that $\lim_{\alpha \to 0}D\left(\alpha ;\xi \right)=\infty$, from the other side, $\lim_{\alpha \to \infty }D\left(\alpha ;\xi \right)=\sum_{i=1}^{n}\log\left[\sin\left(\left(\pi /2\right)G\left(x_{i};\xi \right)\right]\right)<0$, thus, D(α;ξ) is a decreasing function runs from positive to negative. Hence, D(α;ξ)=0 has a unique real root since ∂L(θ)/∂α is negative.  □

By applying the plug-in technique, we can use the estimates of the model parameters to provide estimates of the unknown distribution functions (PDF, SF, etc.).

For an in-depth statistical treatment on the parameters (interval estimations, test procedures…), the observed information matrix is required. Here, it is given by $I\left(\theta \right)=-\partial^{2}L\left(\theta \right)/\left(\partial \theta \partial \theta^{T}\right)$. Now, assume that ξ is a vector of m−1 components, say $\xi ={\left(\xi_{2},\xi_{3},\ldots ,\xi_{m}\right)}^{T}$, and write $\hat{\xi }={\left({\hat{\xi }}_{2},{\hat{\xi }}_{3},\ldots ,{\hat{\xi }}_{m}\right)}^{T}$. Then, the approximate distribution of the random version of $\hat{\theta }$ is $N_{m}\left(\theta ,I{\left(\hat{\theta }\right)}^{-1}\right)$ under the usual regularity conditions. The asymptotic CI for each parameter θ can be determine using 100(1−ϵ)% CI as $ACI_{\alpha }=\left(\hat{\alpha }-\Omega_{\epsilon /2}T_{11},\hat{\alpha }+\Omega_{\epsilon /2}T_{11}\right)$, and $ACI_{\xi_{k}}=\left(\hat{\xi_{k}}-\Omega_{\epsilon /2}T_{kk},\hat{\xi_{k}}+\Omega_{\epsilon /2}T_{kk}\right)$ for 2≤k≤m, where $T_{rr}$ is defined by the square root of the *r*th diagonal component of $I{\left(\hat{\theta }\right)}^{-1})$, for r=1,2,…m and $\Omega_{\epsilon /2}$ is the quantile (1−ϵ/2) of the $N_{1}\left(0,1\right)$ distribution. As a mathematical complement, the components of I(θ) are

$$\begin{array}{cc} I_{\alpha \alpha } & =\frac{n}{\alpha^{2}},\\ I_{\alpha \xi } & =-\frac{\pi }{2}\sum_{i=1}^{n}G^{\xi^{\prime }}\left(x_{i};\xi \right)\cot\left(\frac{\pi }{2}G\left(x_{i};\xi \right)\right),\\ I_{\xi \xi } & =-\sum_{i=1}^{n}\frac{g^{\xi^{\prime }}\left(x_{i};\xi \right)}{g(x_{i};\xi )}+\sum_{i=1}^{n}\frac{{\left(g^{\xi^{\prime }}\left(x_{i};\xi \right)\right)}^{2}}{g^{2}\left(x_{i};\xi \right)}+{\left(\frac{\pi }{2}\right)}^{2}\sum_{i=1}^{n}{\left(G^{\xi^{\prime }}\left(x_{i};\xi \right)\right)}^{2}\sec^{2}\left(\frac{\pi }{2}G\left(x_{i};\xi \right)\right)\\ \; & +\frac{\pi }{2}\sum_{i=1}^{n}G^{\xi^{\prime \prime }}\left(x_{i};\xi \right)\tan\left(\frac{\pi }{2}G\left(x_{i};\xi \right)\right)+\left(\alpha -1\right){\left(\frac{\pi }{2}\right)}^{2}\sum_{i=1}^{n}{\left(G^{\xi^{\prime }}\left(x_{i};\xi \right)\right)}^{2}\csc^{2}\left(\frac{\pi }{2}G\left(x_{i};\xi \right)\right)\\ \; & -\left(\alpha -1\right)\frac{\pi }{2}\sum_{i=1}^{n}G^{\xi^{\prime \prime }}\left(x_{i};\xi \right)\cot\left(\frac{\pi }{2}G\left(x_{i};\xi \right)\right), \end{array}$$

where $g^{\xi^{\prime \prime }}\left(x_{i};\xi \right)=\partial^{2}g\left(x_{i};\xi \right)/\left(\partial \xi \partial \xi^{T}\right)$ and $G^{\xi^{\prime \prime }}\left(x_{i};\xi \right)=\partial^{2}G\left(x_{i};\xi \right)/\left(\partial \xi \partial \xi^{T}\right)$.

### 5.2. Bayes Estimation

In this subsection, we discuss the Bayes estimation of the parameters of the ESW distribution. In this setting, let $\theta ={\left(\alpha ,\beta ,\lambda \right)}^{T}$. The Bayes estimate (BE) vector $\hat{\theta }={\left(\hat{\alpha },\hat{\beta },\hat{\lambda }\right)}^{T}$ is constructed using the posterior distributions given the sample data. The procedure is briefly described below. Let *N* be the number of iterations and *K* be the number of the burn in. Then the square error loss (SEL) function for the assumed prior distribution is given as $S\left(\hat{\theta },\theta \right)={\left(\hat{\theta }-\theta \right)}^{2}$, and is minimized by the posterior mean, as $\hat{\theta }=\left[1/\left(N-K\right)\right]\sum_{i=K+1}^{N}{\hat{\theta }}^{\left(i\right)}$. The highest posterior density (HPD) credible interval for $\hat{\theta }$ is determined using the package HDInterval (see [33]) in the R software. let $X_{1},X_{2},\ldots ,X_{n}$, n≥1, be a sample of independent RVs with the ESW distribution, and $x_{1},x_{2},\ldots ,x_{n}$ be the observations. We recall that the unknown parameters are α,β, and λ. Adopting the Bayesian paradigm, we suppose that α,β, and λ are independent RVs that follow the gamma distribution with PDF defined by

$$p_{i}\left(x\right)=\frac{\vartheta_{i}^{\kappa_{i}}}{\Gamma (\kappa_{i})}x^{\kappa_{i}-1}e^{-\vartheta_{i}x},\;x>0,i=1,2,3,$$

respectively, i.e., $p_{1}\left(x\right)$, $p_{2}\left(x\right)$, and $p_{3}\left(x\right)$ are the prior PDFs. Let L(θ|data) be the likelihood function defined by

$$\begin{array}{cc} L(\theta |data) & ={\left(\frac{\pi }{2}\right)}^{n}\alpha^{n}\beta^{n}\lambda^{n}\prod_{i=1}^{n}x_{i}^{\beta -1}e^{-\lambda \sum_{i=1}^{n}x_{i}^{\beta }}\prod_{i=1}^{n}\cos\left(\frac{\pi }{2}\left(1-e^{-\lambda x_{i}^{\beta }}\right)\right)\\ \; & \times \prod_{i=1}^{n}{\left[\sin\left(\frac{\pi }{2}\left(1-e^{-\lambda x_{i}^{\beta }}\right)\right)\right]}^{\alpha -1}. \end{array}$$

Then, the joint posterior PDF of θ|data is specified by

(22) $$\begin{array}{c} \pi \left(\theta |data\right)=\frac{L\left(\theta |data\right)p_{1}\left(\alpha \right)p_{2}\left(\beta \right)p_{3}\left(\lambda \right)}{\int \int \int g(data;\theta )d\alpha d\beta d\lambda }, \end{array}$$

where $g\left(data;\theta \right)=L\left(\theta |data\right)p_{1}\left(\alpha \right)p_{2}\left(\beta \right)p_{3}\left(\lambda \right)$ is the joint PDF associated with the data. The marginal posterior PDF of α,β, and λ can be derived from Equation (22) as

$$\begin{array}{cc} \pi_{1}\left(\alpha \right) & \propto \alpha^{n+\kappa_{1}-1}e^{-\vartheta_{1}\alpha }\prod_{i=1}^{n}{\left[\sin\left(\frac{\pi }{2}\left(1-e^{-\lambda x_{i}^{\beta }}\right)\right)\right]}^{\alpha -1}, \end{array}$$

$$\begin{array}{cc} \pi_{2}\left(\beta \right) & \propto \beta^{n+\kappa_{2}-1}e^{-\vartheta_{2}\beta -\lambda \sum_{i=1}^{n}x_{i}^{\beta }}\prod_{i=1}^{n}x_{i}^{\beta -1}\prod_{i=1}^{n}\cos\left(\frac{\pi }{2}\left(1-e^{-\lambda x_{i}^{\beta }}\right)\right)\times \\ \; & \prod_{i=1}^{n}{\left[\sin\left(\frac{\pi }{2}\left(1-e^{-\lambda x_{i}^{\beta }}\right)\right)\right]}^{\alpha -1} \end{array}$$

and

$$\begin{array}{cc} \pi_{3}\left(\lambda \right) & \propto \lambda^{n+\kappa_{3}-1}e^{-\lambda \left(\vartheta_{3}+\sum_{i=1}^{n}x_{i}^{\beta }\right)}\prod_{i=1}^{n}\cos\left(\frac{\pi }{2}\left(1-e^{-\lambda x_{i}^{\beta }}\right)\right)\prod_{i=1}^{n}{\left[\sin\left(\frac{\pi }{2}\left(1-e^{-\lambda x_{i}^{\beta }}\right)\right)\right]}^{\alpha -1}. \end{array}$$

The related posterior distributions are not from well-known distributions. As a result, we can obtain samples from posterior distributions using the Metropolis–Hastings (MH) method and the Gibbs sampling technique, as detailed below (see [34,35,36]) (the normal distribution is utilized as a suitable distribution for the MH method):

1. Start by initial guess $\left(\alpha^{\left(0\right)},\beta^{\left(0\right)},\lambda^{\left(0\right)}\right)$,
2. Set t=1,
3. Apply the MH algorithm to generate $\alpha^{\left(t\right)}$ from $\pi_{1}\left(\alpha \right)$,
4. Apply the MH algorithm to generate $\lambda^{\left(t\right)}$ from $\pi_{3}\left(\lambda \right)$,
5. Apply the MH algorithm to generate $\beta^{\left(t\right)}$ from $\pi_{2}\left(\beta \right)$,
6. Set t=t+1,
7. Repeat step 3 to 6, *T* times.

For a very large value of *T*, the estimated vector value of θ can be obtained based on the SEL, and also, the HPD credible interval can be constructed. An approximate 100(1−ϵ)% HPD credible interval of θ can be computed using the idea derived by [37] as the smallest length of the intervals for each parameter $\left({\hat{\theta }}^{1},{\hat{\theta }}^{(1-\epsilon )T}\right),\left({\hat{\theta }}^{2},{\hat{\theta }}^{\left(1-\epsilon )(T+1\right)}\right),\ldots ,\left({\hat{\theta }}^{\epsilon T},{\hat{\theta }}^{T}\right)$.

### 5.3. Simulation Study I

**Figure 4:**
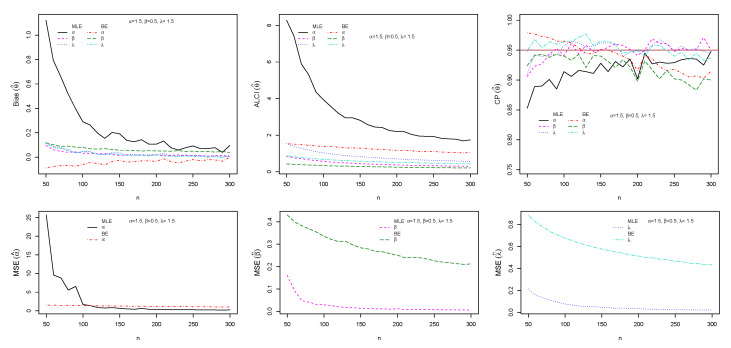
Plots of the Bias, MSE, ALCI, and CP for the estimated α=1.5, β=0.5 and λ=1.5.

**Figure 5:**
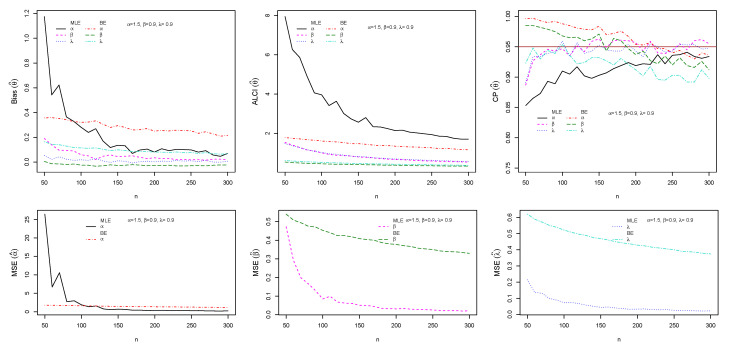
Plots of the Bias, MSE, ALCI, and CP for the estimated α=1.5, β=0.9 and λ=0.9.

**Figure 6:**
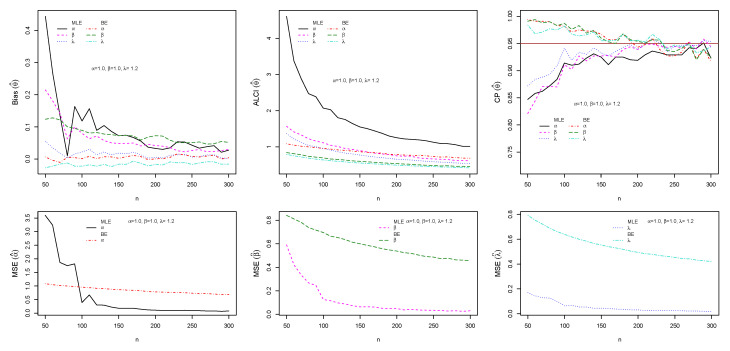
Plots of the Bias, MSE, ALCI, and CP for the estimated α=0.8, β=0.9 and λ=0.5.

**Figure 7:**
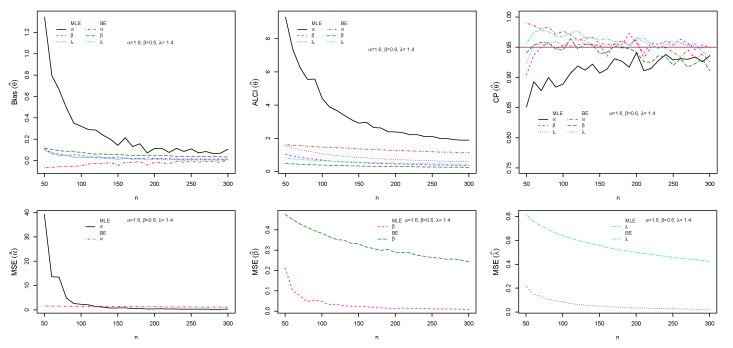
Plots of the Bias, MSE, ALCI, and CP for the estimated α=1.6, β=0.6 and λ=1.4.

The performance of maximum likelihood and Bayes estimations are evaluated using simulation results. We generate 1000 samples of size n=(50,60,70,…,300) from the ESW distribution for some selected parameter values using Equation (17). The bias, mean square error (MSE), the average length of CI (ALCI), and coverage probability (CP) of the estimates are computed using R3.5.3 developed by [38]. For the MLEs, we use the package called maxLik developed by [39] in R-software by maxBFGS. This package allows us to obtain the MLEs and information matrix, and the package matlib elaborated by [40] can be used to get the inverse of the information matrix to compute the confidence interval. The resulting simulations are given in Figure 4, Figure 5, Figure 6 and Figure 7. For the Bayes estimation, we used N= 3000 number of iterations and the first 30% as a burn-in sample. We notice that the estimation behaves well when the all hyperparameters are greater than one and unimodal gamma PDF. It is indicated that the MLE and BE perform consistently, the bias, MSE, and the ALCI decrease as the sample size increases; the CP for each parameter is approaching 0.95 in all cases.

### 5.4. Estimation of the Stress-Strength Reliability from the ESW Distribution

In this part, the expression of a reliability parameter of the ESW distribution is derived when the parameter λ is common. The maximum likelihood estimation of *R* is obtained. The nonparametric percentile bootstrap (Bp) and Student’s bootstrap (Bt) are used to approximate the CI of *R*. Finally, we assessed the estimates through simulation studies.

In the setting of the ESW distribution, let *X* be an RV having the PDF $f(x;\alpha_{1},\beta_{1},\lambda )$ and *Y* be an RV with the CDF $F(x;\alpha_{2},\beta_{2},\lambda )$. We assume that *X* and *Y* are independent. Then, the stress-strength reliability parameter is given by

$$\begin{array}{cc} R & =\int_{-\infty }^{\infty }f\left(x;\alpha_{1},\beta_{1},\lambda \right)F\left(x;\alpha_{2},\beta_{2},\lambda \right)dx\\ \; & =\frac{\pi }{2}\alpha_{1}\beta_{1}\lambda \int_{0}^{\infty }x^{\beta_{1}-1}e^{-\lambda x^{\beta_{1}}}\cos\left(\frac{\pi }{2}\left(1-e^{-\lambda x^{\beta_{1}}}\right)\right){\left[\sin\left(\frac{\pi }{2}\left(1-e^{-\lambda x^{\beta_{1}}}\right)\right)\right]}^{\alpha_{1}-1}\\ \; & \times {\left[\sin\left(\frac{\pi }{2}\left(1-e^{-\lambda x^{\beta_{2}}}\right)\right)\right]}^{\alpha_{2}}dx. \end{array}$$

Now, by considering the changes of variables $u=\left(\pi /2\right)\left(1-e^{-\lambda x^{\beta_{1}}}\right)$ and

$r_{u}=\left(\pi /2\right)\left(1-e^{-\lambda {\left[-\left(1/\lambda \right)\log\left(1-2u/\pi \right)\right]}^{\beta_{2}/\beta_{1}}}\right)$, we get

(23) $$\begin{array}{cc} R & =\alpha_{1}\int_{0}^{\pi /2}\cos u{\left(\sin u\right)}^{\alpha_{1}-1}{\left[\sin(r_{u})\right]}^{\alpha_{2}}du. \end{array}$$

The parameter *R* above has no closed form expression, but it can be easily calculated using mathematical software such as R and Matlab, among others. Moreover, when $\beta_{1}=\beta_{2}$, the expression of *R* can be deduced from Equation (10).

#### 5.4.1. Maximum Likelihood Estimation of *R*

Assume that $X_{1},X_{2},\ldots ,X_{n}$, n≥1, is a sample of independent RVs whose distribution is the $ESW(\alpha_{1},\beta_{1},\lambda )$ distribution, and $Y_{1},Y_{2},\ldots ,Y_{m}$, m≥1, is a sample of independent RVs whose distribution is the $ESW(\alpha_{2},\beta_{2},\lambda )$ distribution. We work with the observations of these samples, denotes by $x_{1},x_{2},\ldots ,x_{n}$ and $y_{1},y_{2},\ldots ,y_{m}$, respectively. Let $\theta ={\left(\alpha_{1},\alpha_{2},\beta_{1},\beta_{2},\lambda \right)}^{T}$ be the vector of unknown parameters, $\hat{\theta }={\left({\hat{\alpha }}_{1},{\hat{\alpha }}_{2},{\hat{\beta }}_{1},{\hat{\beta }}_{2},\hat{\lambda }\right)}^{T}$ be the MLE of θ, and $\hat{R}$ be the estimate of *R*. Then, the log likelihood function is given as

$$\begin{array}{cc} \; & L_{R}\left(\theta \right)=\\ \; & \left(m+n\right)\log\left(\frac{\pi }{2}\right)+n\log\alpha_{1}+n\log\beta_{1}+\left(m+n\right)\log\lambda +\left(\beta_{1}-1\right)\sum_{i=1}^{n}\log x_{i}-\lambda \sum_{i=1}^{n}x_{i}^{\beta_{1}}\\ \; & +\sum_{i=1}^{n}\log\left[\cos\left(\frac{\pi }{2}\left(1-e^{-\lambda x_{i}^{\beta_{1}}}\right)\right)\right]+\left(\alpha_{1}-1\right)\sum_{i=1}^{n}\log\left[\sin\left(\frac{\pi }{2}\left(1-e^{-\lambda x_{i}^{\beta_{1}}}\right)\right)\right]\\ \; & +m\log\alpha_{2}+m\log\beta_{2}+\left(\beta_{2}-1\right)\sum_{j=1}^{m}\log y_{j}-\lambda \sum_{j=1}^{m}y_{j}^{\beta_{2}}\\ \; & +\sum_{j=1}^{m}\log\left[\cos\left(\frac{\pi }{2}\left(1-e^{-\lambda y_{j}^{\beta_{2}}}\right)\right)\right]+\left(\alpha_{2}-1\right)\sum_{j=1}^{m}\log\left[\sin\left(\frac{\pi }{2}\left(1-e^{-\lambda y_{j}^{\beta_{2}}}\right)\right)\right]. \end{array}$$

It can be maximized with respect to θ to obtain $\hat{\theta }$. The above equation can equivalently be maximized by the solution of the system of equations given below:

$$\begin{array}{cc} \frac{\partial L_{R}\left(\theta \right)}{\partial \alpha_{1}} & =\frac{n}{\alpha_{1}}+\sum_{i=1}^{n}\log\left[\sin\left(\frac{\pi }{2}\left(1-e^{-\lambda x_{i}^{\beta_{1}}}\right)\right]\right)=0, \end{array}$$

$$\begin{array}{cc} \frac{\partial L_{R}\left(\theta \right)}{\partial \alpha_{2}} & =\frac{m}{\alpha_{2}}+\sum_{j=1}^{m}\log\left[\sin\left(\frac{\pi }{2}\left(1-e^{-\lambda y_{j}^{\beta_{2}}}\right)\right)\right]=0, \end{array}$$

$$\begin{array}{cc} \frac{\partial L_{R}\left(\theta \right)}{\partial \beta_{1}} & =\frac{n}{\beta_{1}}+\sum_{i=1}^{n}\log x_{i}-\lambda \sum_{i=1}^{n}x_{i}^{\beta_{1}}\log x_{i}\\ \; & -\lambda \frac{\pi }{2}\sum_{i=1}^{n}\log\left[\tan\left(\frac{\pi }{2}\left(1-e^{-\lambda x_{i}^{\beta_{1}}}\right)\right)\right]e^{-\lambda x_{i}^{\beta_{1}}}x_{i}^{\beta_{1}}\log x_{i}\\ \; & +\left(\alpha_{1}-1\right)\lambda \frac{\pi }{2}\sum_{i=1}^{n}\log\left[\cot\left(\frac{\pi }{2}\left(1-e^{-\lambda x_{i}^{\beta_{1}}}\right)\right)\right]e^{-\lambda x_{i}^{\beta_{1}}}x_{i}^{\beta_{1}}\log x_{i}=0, \end{array}$$

$$\begin{array}{cc} \frac{\partial L_{R}\left(\theta \right)}{\partial \beta_{2}} & =\frac{m}{\beta_{2}}+\sum_{j=1}^{m}\log y_{j}-\lambda \sum_{j=1}^{m}y_{j}^{\beta_{2}}\log y_{j}\\ \; & -\lambda \frac{\pi }{2}\sum_{j=1}^{m}\log\left[\tan\left(\frac{\pi }{2}\left(1-e^{-\lambda y_{j}^{\beta_{2}}}\right)\right)\right]e^{-\lambda y_{j}^{\beta_{2}}}y_{j}^{\beta_{2}}\log y_{j}\\ \; & +\left(\alpha_{2}-1\right)\lambda \frac{\pi }{2}\sum_{j=1}^{m}\log\left[\cot\left(\frac{\pi }{2}\left(1-e^{-\lambda y_{j}^{\beta_{2}}}\right)\right)\right]e^{-\lambda y_{j}^{\beta_{2}}}y_{j}^{\beta_{2}}\log y_{j}=0 \end{array}$$

and

$$\begin{array}{cc} \frac{\partial L_{R}\left(\theta \right)}{\partial \beta_{2}} & =\frac{m+n}{\lambda }-\sum_{i=1}^{n}x_{i}^{\beta_{1}}-\lambda \sum_{j=1}^{m}y_{j}^{\beta_{2}}\\ \; & -\frac{\pi }{2}\sum_{i=1}^{n}\log\left[\tan\left(\frac{\pi }{2}\left(1-e^{-\lambda x_{i}^{\beta_{1}}}\right)\right)\right]e^{-\lambda x_{i}^{\beta_{1}}}x_{i}^{\beta_{1}}\\ \; & -\frac{\pi }{2}\sum_{j=1}^{m}\log\left[\tan\left(\frac{\pi }{2}\left(1-e^{-\lambda y_{j}^{\beta_{2}}}\right)\right)\right]e^{-\lambda y_{j}^{\beta_{2}}}y_{j}^{\beta_{2}}\\ \; & +\left(\alpha_{1}-1\right)\frac{\pi }{2}\sum_{i=1}^{n}\log\left[\cot\left(\frac{\pi }{2}\left(1-e^{-\lambda x_{i}^{\beta_{1}}}\right)\right)\right]e^{-\lambda x_{i}^{\beta_{1}}}x_{i}^{\beta_{1}}\\ \; & +\left(\alpha_{2}-1\right)\frac{\pi }{2}\sum_{j=1}^{m}\log\left[\cot\left(\frac{\pi }{2}\left(1-e^{-\lambda y_{j}^{\beta_{2}}}\right)\right)\right]e^{-\lambda y_{j}^{\beta_{2}}}y_{j}^{\beta_{2}}=0. \end{array}$$

Once the MLE $\hat{\theta }$ is obtained, we can compute $\hat{R}$ from Equation (23).

#### 5.4.2. Bootstrap CIs for *R*

Here, we propose the use of two nonparametric CI, the percentile Bp CI, and the Bt CI, as discussed in [41]. The following steps are required to obtain the bootstrap CIs:

1. We generate a sample of values $x_{1},x_{2},x_{3},\ldots ,x_{n}$ from the $ESW(\alpha_{1},\beta_{1},\lambda )$ distribution, and an independent sample of values $y_{1},y_{2},y_{3},\ldots ,y_{m}$ from the $ESW(\alpha_{2},\beta_{2},\lambda )$ distribution.
2. We generate independent bootstrap samples of values $x_{1}^{*},x_{2}^{*},x_{3}^{*},\ldots ,x_{n}^{*}$ and $y_{1}^{*},y_{2}^{*},y_{3}^{*},\ldots ,y_{m}^{*}$ using sampling with replacement from the first step in above. Based on the bootstrap sample, we compute the MLE of θ, say ${\hat{\theta }}^{*}={\left({\hat{\alpha }}_{1}^{*},{\hat{\alpha }}_{2}^{*},{\hat{\beta }}_{1}^{*},{\hat{\beta }}_{2}^{*},{\hat{\lambda }}^{*}\right)}^{T}$, then compute the corresponding MLE of *R*, say ${\hat{R}}^{*}$.
3. In order to get a set of bootstrap samples of *R*, repeat step 2 to 3 B-times. We consider the samples ordered in an increasing order, say ${\hat{R}}_{j}^{*}$, j=1,2,…,B.

Then, bootstrap CIs of *R* can be obtained.

- *Percentile bootstrap CI:*
  Let ${\hat{R}}_{\left(\delta \right)}^{*}$ be the δ percentile of ${\hat{R}}_{j}^{*}$, j=1,2,3,…,B. That is
  $$\begin{array}{c} \frac{1}{B}\sum_{j=1}^{B}I_{\left\{{\hat{R}}_{j}^{*}\leq {\hat{R}}_{\delta }^{*}\right\}}=\delta ,\;0<\delta <1, \end{array}$$
  where $I_{\left\{.\right\}}$ denotes the standard indicator function. A 100(1−ϵ)% Bp CI of *R* is given as
  $$\begin{array}{c} \left({\hat{R}}_{\left(\epsilon /2\right)}^{*},{\hat{R}}_{\left(1-\epsilon /2\right)}^{*}\right). \end{array}$$
- *Student’s t bootstrap CI:*
  Let us set
  $$\begin{array}{c} {\bar{\hat{R}}}^{*}=\frac{1}{B}\sum_{j=1}^{B}{\hat{R}}_{j}^{*},\;se\left({\hat{R}}^{*}\right)=\sqrt{\frac{1}{B}\sum_{j=1}^{B}{\left({\hat{R}}_{j}^{*}-{\bar{\hat{R}}}^{*}\right)}^{2}}, \end{array}$$
  and ${\hat{t}}_{\delta }^{*}$ be the δ percentile of $\left({\hat{R}}_{j}^{*}-\hat{R}\right)/se({\hat{R}}^{*})$, j=1,2,…,3, such that
  $$\begin{array}{c} \frac{1}{B}\sum_{j=1}^{B}I_{\left({\hat{R}}_{j}^{*}-\hat{R}\right)/se({\hat{R}}^{*})\leq {\hat{t}}_{\delta }^{*}}=\delta ,\;0<\tau <1. \end{array}$$
  With these tools, a 100(1−ϵ)%$B_{t}$ CI of *R* is given as
  $$\begin{array}{c} {\bar{\hat{R}}}^{*}\pm {\hat{t}}_{\left(\epsilon /2\right)}^{*}se\left({\hat{R}}^{*}\right)=\left({\bar{\hat{R}}}^{*}-{\hat{t}}_{\left(\epsilon /2\right)}^{*}se\left({\hat{R}}^{*}\right),{\bar{\hat{R}}}^{*}+{\hat{t}}_{\left(\epsilon /2\right)}^{*}se\left({\hat{R}}^{*}\right)\right). \end{array}$$

#### 5.4.3. Simulation Study II

Here, a simulation is conducted to study the performance of the MLE and bootstrap CI of *R*. A simulated sample size of 1000 is obtained via Equation (17) for various sample sizes and parameters from the $ESW(\alpha_{1},\beta_{1},\lambda )$ and $ESW(\alpha_{2},\beta_{2},\lambda )$ distributions. Let *n* and *m* be these sample sizes, respectively. We consider various cases of (n,m) as (20,20),(30,20),(40,40),(40,60) and (60,60). In each case, the MLEs of *R* is obtained and the standard deviation (SD), and bias and MSE of *R* are considered. A 95% CI of *R* is obtained using the nonparametric percentile Bp and Bt CIs. The bootstrap is obtained using B=1000 replications. The result of the simulation studies is given in Table 1. It shows that, as the two sample sizes increase, the SDs and MSEs decrease; the estimated value of *R* converges to its actual value; the ALCI in the two bootstrap methods decreases as the sample size increases. Also, the CP goes to the nominal level of 0.95.

## 6. Application

Using three real data applications, we demonstrate the performance of the ESW model in comparison to its submodels and several other existing models. Two data sets for stress-strength reliability assessments make up one application. We estimate all the competing model’s parameters by the maximum likelihood method. Also, the parameters of the ESW model are further estimated by the Bayes techniques. We traditionally compare the fitted models using the Akaike information criterion (AIC), Bayesian information criterion (BIC), and consistent Akaike information criterion (CAIC), all are defined via the estimated log-likelihood (L) as a main ingredient. Further, the goodness-of-fit statistics known as the Kolmogorov-Smirnov (KS), Anderson-Darling (AD), and Cramér-von Mises (CvM) are considered. As usual, the data are better represented by the model with the least value of these metrics than the other models. In the case of the MLEs and BEs of the parameters of the ESW model, we compare them via the KS, AD, and CvM. The competing models are generalized exponential (GE) model (see [42]), generalized Rayleigh (GR) model (see [43]), generalized exponential Poisson (GEP) model (see [44]), half logistic Poisson (HLP) model (see [45]), exponentiated Nadarajah-Haghighi (ENH) model (see [46,47]), generalized inverse Weibull (GIW) model (see [48]), and W model.

### 6.1. Real Data Application I

This data set is constituted by the total milk production from the first birth of 107 cows of the SINDI race. The data can be found in [49]. They were also analyzed by [10]. Concretely, the data set is: 0.4365, 0.4260, 0.5140, 0.6907, 0.7471, 0.2605, 0.6196, 0.8781, 0.4990, 0.6058, 0.6891, 0.5770, 0.5394, 0.1479, 0.2356, 0.6012, 0.1525, 0.5483, 0.6927, 0.7261, 0.3323, 0.0671, 0.2361, 0.4800, 0.5707, 0.7131, 0.5853, 0.6768, 0.5350, 0.4151, 0.6789, 0.4576, 0.3259, 0.2303, 0.7687, 0.4371, 0.3383, 0.6114, 0.3480, 0.4564, 0.7804, 0.3406, 0.4823, 0.5912, 0.5744, 0.5481, 0.1131, 0.7290, 0.0168, 0.5529, 0.4530, 0.3891, 0.4752, 0.3134, 0.3175, 0.1167, 0.6750, 0.5113, 0.5447, 0.4143, 0.5627, 0.5150, 0.0776, 0.3945, 0.4553, 0.4470, 0.5285, 0.5232, 0.6465, 0.0650, 0.8492, 0.8147, 0.3627, 0.3906, 0.4438, 0.4612, 0.3188, 0.2160, 0.6707, 0.6220, 0.5629, 0.4675, 0.6844, 0.3413, 0.4332, 0.0854, 0.3821, 0.4694, 0.3635, 0.4111, 0.5349, 0.3751, 0.1546, 0.4517, 0.2681, 0.4049, 0.5553, 0.5878, 0.4741, 0.3598, 0.7629, 0.5941, 0.6174, 0.6860, 0.0609, 0.6488, 0.2747.

**Figure 8:**
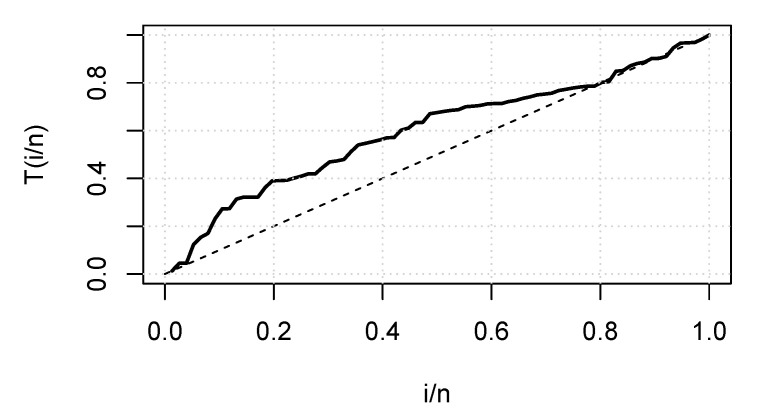
TTT plot for the data I.

**Figure 9:**
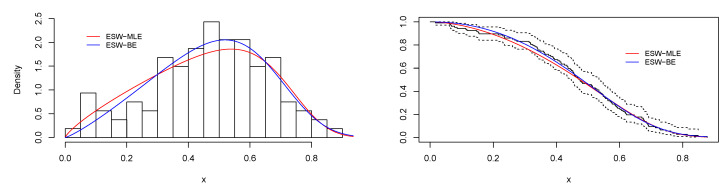
Plots of histogram with fitted PDF of the ESW model, and empirical SF with fitted SF of the ESW model for the data I.

**Figure 10:**
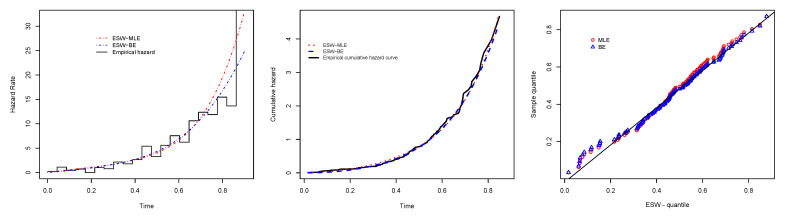
Plots of empirical with fitted HRF of the ESW model, empirical CHRF with fitted CHRF of the ESW model, and QQ plot for the data I.

**Figure 11:**
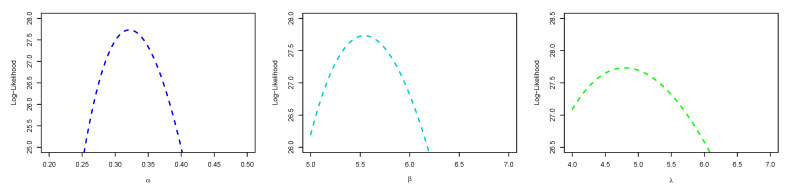
Plots of profile log-likelihood function of each parameter for the data I.

**Figure 12:**
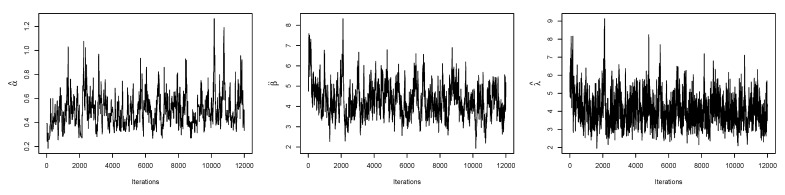
Plots of iterations from the MH algorithm and Gibbs sampling technique for the data I.

**Figure 13:**
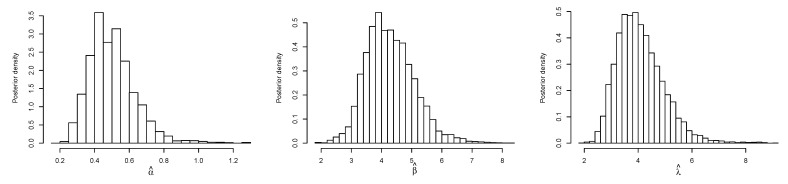
Plots of posterior PDFs of α,β and λ in the ESW model based on iterations for the data I.

Figure 8 is the total time on test (TTT) plot of the data set. This shows that data exhibiting an increasing HRF. The ESW model is a capable candidate to accommodate this kind of HRF. The numerical values of the considered statistical measures for each model are computed and presented in Table 2. The results show that the ESW model provides a better fit than the other competing models. The Bayes estimation method shows a better fit in terms of KS, while the maximum likelihood method shows a better fit in terms of AD and CvM. Both of these techniques are sufficient choices for parameter estimation of the ESW model. Figure 9 illustrates the plots of the histogram with the fitted PDF of the ESW model and the empirical SF with the fitted SF of the ESW model. Figure 10 displays the plots of the HRF of the ESW model for the data; empirical cumulative HRF (CHRF) with the fitted CHRF of the ESW model, and quantile-quantile (QQ) plot of the ESW model. The obtained fits are totally satisfying. Figure 11 illustrates the profile log-likelihood of each parameter for the given data set. The uniqueness of the estimates can be seen. To show the performance of the BEs, Figure 12 shows the iterations obtained from the MH algorithm and the Gibbs sampling technique for each parameter, and Figure 13 shows the posterior PDFs of each parameter based on the iterations.

We end this part by indicating the observed information matrix of the ESW model:

$$I\left(\hat{\alpha },\hat{\beta },\hat{\lambda }\right)=\left(\begin{array}{ccc} 1034.53601 & 90.1359 & -19.22373\\ 90.13590 & -107836.9429 & -107846.01301\\ -19.22373 & -107846.0130 & -107775.44190 \end{array}\right)$$

and

$$I{\left(\hat{\alpha },\hat{\beta },\hat{\lambda }\right)}^{-1}=\left(\begin{array}{ccc} 0.00113068 & -0.00155187 & 0.00155269\\ -0.00155187 & 0.01467901 & -0.01468834\\ 0.00155269 & -0.01468834 & 0.01468841 \end{array}\right).$$

### 6.2. Real Data Application II

This data set is provided by [50]. It consists of the remission times (in months) of a random sample of 128 bladder cancer patients, also studied by [25,51]. The data set is: 0.08, 2.09, 3.48, 4.87, 6.94, 8.66, 13.11, 23.63, 0.20, 2.23, 3.52, 4.98, 6.97, 9.02, 13.29, 0.40, 2.26, 3.57, 5.06, 7.09, 9.22, 13.80, 25.74, 0.50, 2.46, 3.64, 5.09, 7.26, 9.47, 14.24, 25.82, 0.51, 2.54, 3.70, 5.17, 7.28, 9.74, 14.76, 26.31, 0.81, 2.62, 3.82, 5.32, 7.32, 10.06, 14.77, 32.15, 2.64, 3.88, 5.32, 7.39, 10.34, 14.83, 34.26, 0.90, 2.69, 4.18, 5.34, 7.59, 10.66, 15.96, 36.66, 1.05, 2.69, 4.23, 5.41, 7.62, 10.75, 16.62, 43.01, 1.19, 2.75, 4.26, 5.41, 7.63, 17.12, 46.12, 1.26, 2.83, 4.33, 5.49, 7.66, 11.25, 17.14, 79.05, 1.35, 2.87, 5.62, 7.87, 11.64, 17.36, 1.40, 3.02, 4.34, 5.71, 7.93, 11.79, 18.10, 1.46, 4.40, 5.85, 8.26, 11.98, 19.13, 1.76, 3.25, 4.50, 6.25, 8.37, 12.02, 2.02, 3.31, 4.51, 6.54, 8.53, 12.03, 20.28, 2.02, 3.36, 6.76, 12.07, 21.73, 2.07, 3.36, 6.93, 8.65, 12.63, 22.69.

**Figure 14:**
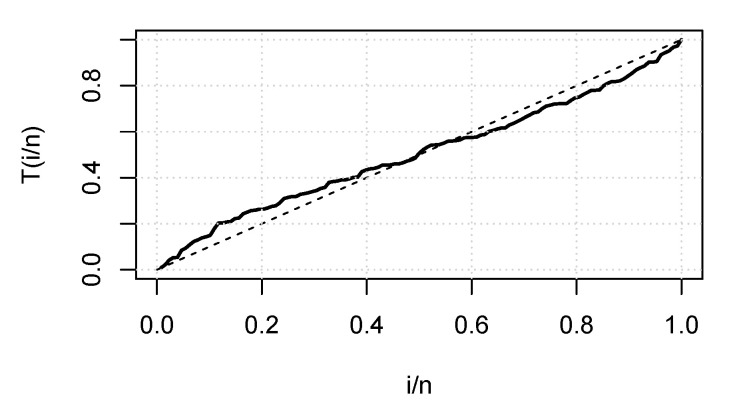
TTT plot for the data II.

**Figure 15:**
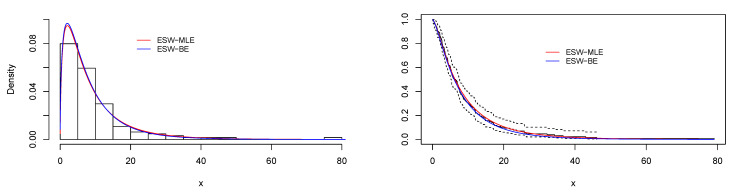
Plots of histogram with the fitted PDF of the ESW model, and empirical SF with fitted SF of the ESW model for the data II.

**Figure 16:**
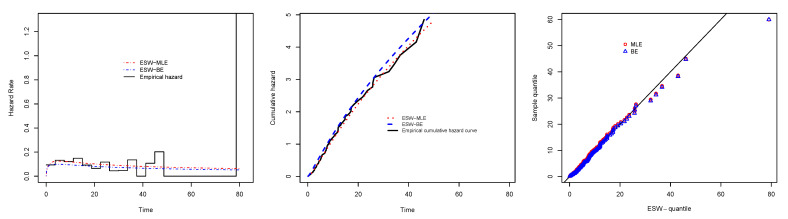
Plots of empirical HRF with fitted HRF of the ESW model, empirical CHRF with fitted CHRF, and QQ plot for the ESW model for the data II.

**Figure 17:**
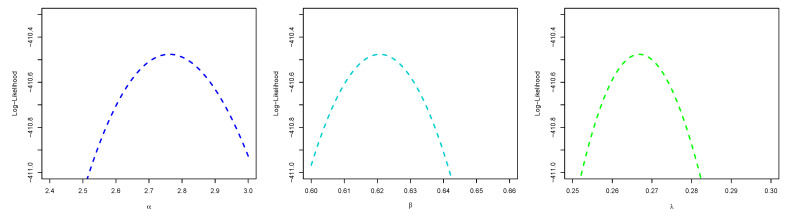
Plots of profile log-likelihood function of each parameter of the ESW model for the data II.

**Figure 18:**
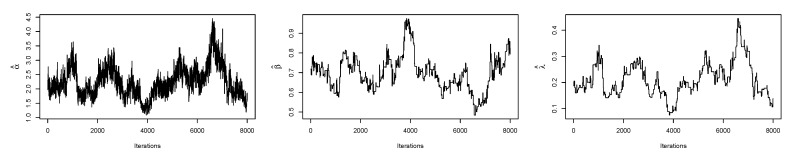
Plots of iterations from the MH algorithm and Gibbs sampling technique for the data II.

**Figure 19:**
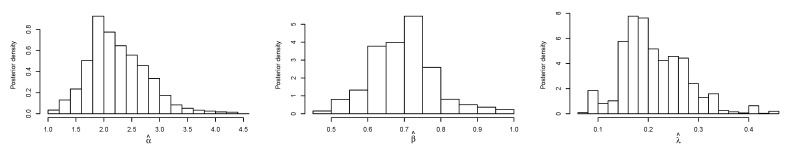
Plots of posterior PDFs of α,β and λ in the ESW model based on iterations for the data II.

Figure 14 displays the TTT plot of the data set. It shows that the data exhibit an upside-down-bathtub HRF, and the ESW model is a capable candidate to accommodate such an HRF. The numerical values of these measures for all the models are computed and given in Table 3; the results show that the ESW model captures the information of the data better than the other competing models. Figure 15 gives the plot of the histogram with the fitted PDF of the ESW model and empirical SF with the fitted SF of the ESW model. Figure 16 shows the plot of the HRF of the ESW model, empirical CHRF with the fitted CHRF of the ESW model, and QQ plot of the ESW model. Figure 17 is the plots of the profile log-likelihood of each parameter. To illustrate the Bayes estimates performance, Figure 18 shows the iterations obtained from the MH algorithm and the Gibbs sampling technique for each parameter, and Figure 19 shows the posterior PDFs of each parameter based on the iterations obtained.

To end this portion, the observed information matrix of the ESW model is computed as

$$I\left(\hat{\alpha },\hat{\beta },\hat{\lambda }\right)=\left(\begin{array}{ccc} 16.71197 & -72.64589 & -220.8935\\ -72.64589 & 2307.67228 & 2847.1163\\ -220.89353 & 2847.11632 & 4766.8891 \end{array}\right)$$

and

$$I{\left(\hat{\alpha },\hat{\beta },\hat{\lambda }\right)}^{-1}=\left(\begin{array}{ccc} 1.4557178 & -0.14214165 & 0.15235355\\ -0.1421417 & 0.01552620 & -0.01586004\\ 0.1523536 & -0.01586004 & 0.01674243 \end{array}\right).$$

### 6.3. Real Data Application III

In this subsection, we illustrate the performance of the ESW model in the stress strength reliability studies by using real data sets. Also, we demonstrate the application of the proposed estimation techniques in a practical scenario. We compute the value of *R* by maximum likelihood approach and the 95% nonparametric Bp and Bt CIs of *R* using B=1000 replications. The KS is used to show how good the fit of the ESW model is for the two data sets.

The reference [52] provides the following data sets. One data set represents single fibers tested under tension at gauge lengths of 10 mm (data1), and the other represents impregnated tows of 1000 fibers tested at gauge lengths of 20 mm (data2), of sizes n=63 and m=69, respectively.

They are given as:

Data1(X): 1.901, 2.132, 2.203, 2.228, 2.257, 2.350, 2.361, 2.396, 2.397, 2.445, 2.454, 2.474, 2.518, 2.522, 2.525, 2.532, 2.575, 2.614, 2.616, 2.618, 2.624, 2.659, 2.675, 2.738, 2.740, 2.856, 2.917, 2.928, 2.937, 2.937, 2.977, 2.996, 3.030, 3.125, 3.139, 3.145, 3.220, 3.223, 3.235, 3.243, 3.264, 3.272, 3.294, 3.332, 3.346, 3.377, 3.408, 3.435, 3.493, 3.501, 3.537, 3.554, 3.562, 3.628, 3.852, 3.871, 3.886, 3.971, 4.024, 4.027, 4.225, 4.395, 5.020.

Data2(Y): 1.312, 1.314, 1.479, 1.552, 1.700, 1.803, 1.861, 1.865, 1.944, 1.958, 1.966, 1.997, 2.006, 2.021, 2.027, 2.055, 2.063, 2.098, 2.14, 2.179, 2.224, 2.240, 2.253, 2.270, 2.272, 2.274, 2.301, 2.301, 2.359, 2.382, 2.382, 2.426, 2.434, 2.435, 2.478, 2.490, 2.511, 2.514, 2.535, 2.554, 2.566, 2.57, 2.586, 2.629, 2.633, 2.642, 2.648, 2.684, 2.697, 2.726, 2.770, 2.773, 2.800, 2.809, 2.818, 2.821, 2.848, 2.88, 2.954, 3.012, 3.067, 3.084, 3.090, 3.096, 3.128, 3.233, 3.433, 3.585, 3.585.

**Figure 20:**
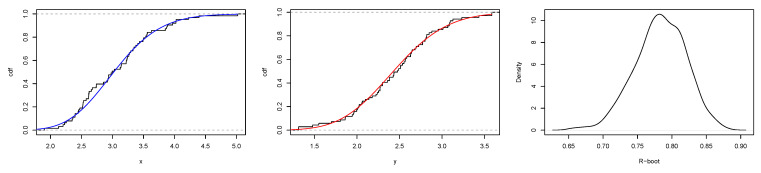
Plots of empirical and fitted CDF of the ESW model, and PDF of estimated bootstrap values of *R* for stress-strength data sets.

**Figure 21:**
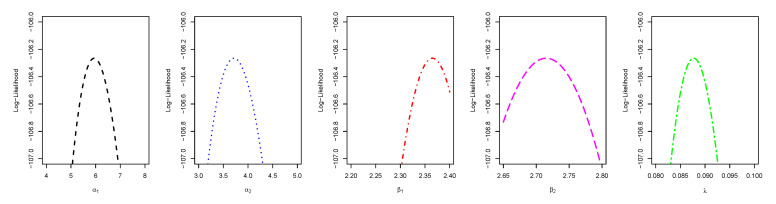
Plots of profile log-likelihood function of each parameter for stress-strength data sets.

The estimated values from the application are given in Table 4; it is quite clear that the ESW model fits the two data sets well as measured by KS (i.e., $KS_{1}$ for the ESW distribution underlying Data1(X) and $KS_{2}$ for the ESW distribution underlying Data2(X)), and its performance indicates that the ESW model can be considered as a good candidate in stress strength reliability analysis. Figure 20 displays the empirical and fitted CDF of the ESW model, and the PDF of the estimated bootstrap values of *R* for the data sets. Figure 21 shows the profile log likelihood of the estimated parameters. It proves that the obtained MLE is unique.

## 7. Conclusions

The exponentiated sine-generated family is a new family of distributions proposed in this paper. Some important mathematical and statistical properties were derived, such as the series representation of the probability density function, quantile function, moments, stress-strength reliability parameter, and Rényi entropy. A special member of the family, called the exponentiated sine Weibull (ESW) distribution, was derived and studied. We analyzed its skewness and kurtosis, mean residual and reversed mean residual life functions, order statistics, and extreme value distributions. The maximum likelihood estimation and Bayes estimation under the square error loss function of the ESW model were discussed. They were assessed using simulation studies via the mean square error and confidence interval of the estimates, and they work well. The expression of a reliability parameter of the ESW distribution is derived when the scale parameter is common. The maximum likelihood is used to estimate this special parameter, and nonparametric bootstrap techniques were considered for the confidence interval. The estimation was assessed by simulation studies and worked well by examining the mean square error, standard deviations, confidence intervals, and coverage probability. In the end, three applications of the ESW model to real data were provided for illustration, in which the ESW model outperforms some other existing models in terms of fitting and is demonstrated as a good candidate for stress-strength parameter analysis. The potential for new applications of the ESW model in the applied field is huge, opening the door to new statistical analysis in applied science dealing with important lifetime data. Also, another specific member of the new family can be investigated for further applied perspectives.

## Figures and Tables

**Table 1:** Parameters’ values, actual value of *R*, estimated value of *R* with SD in parenthesis, MSE of $\hat{R}$ with bias in parenthesis, and ALCI with CP in parenthesis.

$\left(\alpha_{1},\alpha_{2},\beta_{1},\beta_{2},\lambda \right)$	*R*	(n,m)	$\hat{R}\left(\mathrm{SD}\right)$	MSE(Bias)	${\mathrm{ALCI}}_{\mathrm{Bp}}\left(\mathrm{CP}\right)$	${\mathrm{ALCI}}_{\mathrm{Bt}}\left(\mathrm{CP}\right)$
(0.8, 0.7, 0.5, 0.6, 0.5)	0.5182	(20, 20)	0.5187 (0.0939)	0.0088 (0.0006)	0.4153 (0.93)	0.4971 (0.94)
		(30, 20)	0.5233 (0.0778)	0.0061 (0.0052)	0.3238 (0.94)	0.3544 (0.94)
		(40, 40)	0.5191 (0.0585)	0.0034 (0.0010)	0.2323 (0.94)	0.2341 (0.94)
		(40, 60)	0.5181 (0.0544)	0.0030 ($-7\times 10^{-5}$)	0.2115 (0.95)	0.2108 (0.95)
		(60, 60)	0.5189 (0.0496)	0.0025 (0.0007)	0.1871 (0.94)	0.1876 (0.94)
(0.8, 0.7, 1.2, 1.0, 0.6)	0.5555	(20, 20)	0.5080 (0.1827)	0.0356 (−0.0475)	0.6756 (0.94)	0.9333 (0.89)
		(30, 20)	0.5263 (0.1411)	0.0208 (−0.0292)	0.6082 (0.94)	0.8711 (0.92)
		(40, 40)	0.5511 (0.0792)	0.0063 (−0.0044)	0.4348 (0.94)	0.6333 (0.96)
		(40, 60)	0.5546 (0.0665)	0.0044 (−0.0009)	0.3434 (0.94)	0.4769 (0.96)
		(60, 60)	0.5564 (0.0509)	0.0026 (0.0008)	0.2636 (0.95)	0.3413 (0.95)
(0.9, 0.5, 0.6, 0.8, 0.7)	0.5972	(20, 20)	0.5970 (0.0893)	0.0080 (−0.0021)	0.4574 (0.94)	0.5967 (0.95)
		(30, 20)	0.5988 (0.0748)	0.0056 (0.0015)	0.3406 (0.94)	0.4051 (0.95)
		(40, 40)	0.5976 (0.0575)	0.0033 (0.0004)	0.2283 (0.95)	0.2408 (0.95)
		(40, 60)	0.5986 (0.537)	0.0029 (0.0013)	0.2053 (0.93)	0.2081 (0.93)
		(60, 60)	0.5997 (0.0479)	0.0023 (0.0025)	0.1784 (0.94)	0.1793 (0.94)
(0.6, 0.5, 0.9, 0.9, 0.7)	0.5455	(20, 20)	0.5278 (0.1235)	0.0155 (−0.0176)	0.5598 (0.94)	0.7735 (0.95)
		(30, 20)	0.5378 (0.0882)	0.0078 (−0.0076)	0.4385 (0.94)	0.5898 (0.96)
		(40, 40)	0.5467 (0.0618)	0.0038 (0.0012)	0.2780 (0.94)	0.3333 (0.94)
		(40, 60)	0.5441 (0.0555)	0.0031 (−0.0013)	0.2554 (0.94)	0.3065 (0.94)
		(60, 60)	0.5458 (0.0480)	0.0023 (0.0004)	0.1870 (0.93)	0.1940 (0.93)
(2.5, 1.3, 2.0, 1.1, 1.4)	0.7736	(20, 20)	0.7233 (0.2232)	0.05229 (−0.0503)	0.7678 (0.95)	1.1720 (0.88)
		(30, 20)	0.7481 (0.1661)	0.0282 (−0.0256)	0.6759 (0.94)	1.0583 (0.93)
		(40, 40)	0.7594 (0.1162)	0.0137 (−0.0142)	0.4909 (0.95)	0.7720 (0.95)
		(40, 60)	0.7707 (0.0819)	0.0067 (−0.0030)	0.4094 (0.94)	0.6441 (0.93)
		(60, 60)	0.7757 (0.0530)	0.0028 (0.0021)	0.2846 (0.92)	0.4186 (0.93)
(2.5, 0.5, 1.0, 2.6, 0.3)	0.9084	(20, 20)	0.9074 (0.0668)	0.0045 (−0.0010)	0.3645 (0.90)	0.5907 (0.91)
		(30, 20)	0.9073 (0.0627)	0.0039 (−0.0011)	0.2843 (0.92)	0.4423 (0.93)
		(40, 40)	0.9106 (0.0321)	0.0010 (0.0022)	0.1314 (0.90)	0.1574 (0.91)
		(40, 60)	0.9108 (0.0305)	0.0009 (0.0024)	0.1111 (0.90)	0.1204 (0.90)
		(60, 60)	0.9092 (0.0259)	0.0007 (0.0008)	0.0969 (0.93)	0.1029 (0.93)
(3.8, 1.2, 1.5, 1.2, 1.1)	0.7787	(20, 20)	0.7567 (0.1584)	0.0255 (−0.0219)	0.6621 (0.94)	1.0187 (0.92)
		(30, 20)	0.7743 (0.1167)	0.0136 (−0.0044)	0.5471 (0.91)	0.8355 (0.91)
		(40, 40)	0.7594 (0.1163)	0.0137 (−0.0142)	0.4909 (0.95)	0.7720 (0.95)
		(40, 60)	0.7783 (0.0601)	0.0036 (−0.0004)	0.3099 (0.94)	0.4329 (0.95)
		(60, 60)	0.7791 (0.0412)	0.0017 (0.0005)	0.1740 (0.93)	0.1969 (0.93)
(1.0, 1.0, 1.0, 1.0, 1.0)	0.5000	(20, 20)	0.4738 (0.1447)	0.0216 (−0.0262)	0.5975 (0.94)	0.8229 (0.92)
		(30, 20)	0.4854 (0.1195)	0.0145 (−0.0146)	0.5135 (0.94)	0.7150 (0.94)
		(40, 40)	0.4940 (0.0841)	0.0071 (−0.0060)	0.4511 (0.95)	0.6473 (0.96)
		(40, 60)	0.4964 (0.0665)	0.0044 (−0.0036)	0.3099 (0.94)	0.4093 (0.95)
		(60, 60)	0.4993 (0.0464)	0.0022 (−0.0007)	0.2297 (0.94)	0.2780 (0.96)

**Table 2:** MLEs, BE with 95% CI in parenthesis, L, AIC, BIC, CAIC, KS with *p*-value in parenthesis, AD, and CvM for the data I.

Model	α	β	λ	θ	L	AIC	BIC	CAIC	KS	AD	CvM
ESW ${}_{MLE}$	0.3216	5.5424	4.7906	-	27.732	−49.464	−41.446	−49.231	0.0734	0.5305	0.0836
	(0.2557, 0.3875)	(5.3049, 5.7799)	(4.5531, 5.0282)						(0.6113)		
ESW ${}_{BE}$	0.5021	4.2903	4.0846	-	-	-	-	-	0.0573	0.5825	0.0871
	(0.2604, 0.7447)	(2.8145, 5.8670)	(2.6074, 5.7682)						(0.8744)		
ESE	3.3101	-	2.1712	-	6.596	−9.191	−3.846	−9.076	0.1418	4.0970	0.6751
	(2.3421, 4.2782)		(1.8087, 2.5336)						(0.0271)		
SW	-	2.4894	2.8311	-	22.378	−40.754	−35.409	−40.639	0.0766	1.3299	0.2003
		(2.0912, 2.8857)	(2.0695, 3.5926)						(0.5559)		
W	-	2.6012	5.3818	-	21.348	−38.695	−33.349	−38.580	0.0832	1.5126	0.2307
		(2.1896, 3.0128)	(3.8514, 6.9122)						(0.4487)		
GE	3.7139	-	4.2007	-	5.039	−6.078	−0.732	−5.962	0.1477	4.3696	0.7257
	(2.6102, 4.8176)		(3.4729, 4.9284)						(0.0188)		
GR	2.1189	-	-	1.2567	18.155	−32.311	−26.195	−32.195	0.1178	2.1579	0.3371
	(2.6102, 4.8176)			(3.4730, 4.9284)					(0.1026)		
GEP	4.2690	3.7540	4.1128	-	5.0158	−4.0316	3.9869	−3.7986	0.1544	4.3799	0.7276
	(4.2439, 4.2947)	(3.0422, 4.4662)	(0, 0.0061)						(0.0121)		
HLP	5.7570	-	−4.9618	-	17.877	−31.754	−26.409	−31.639	0.0916	1.9932	0.3015
	(5.0236, 6.4923)		(−6.4447, −3.4788)						(0.3302)		
ENH	32.5264	2.3595	0.0608	-	21.0381	−36.076	−28.058	−35.843	0.2995	1.3831	0.2104
	(0, 74.2789)	(1.7901, 2.9288)	(0, 0.1409)						($9.3\times 10^{-9}$)		

**Table 3:** MLEs, BE with 95% CI in parenthesis, L, AIC, BIC, CAIC, KS with *p*-value in parenthesis, AD, and CvM for the data II.

Model	α	β	λ	θ	γ	L	AIC	BIC	CAIC	KS	AD	CvM
ESW ${}_{MLE}$	2.7619	0.6207	0.2668	-	-	−410.476	826.95	835.508	827.146	0.0435	0.2594	0.0394
	(0.3971, 5.1267)	(0.3765, 0.8649)	(0.0132, 0.5204)							(0.9688)		
ESW ${}_{BE}$	2.2475	0.6275	0.2614	-	-	-	-	-	-	0.0507	0.2952	0.0468
	(1.2466, 3.2377)	(0.5272, 0.8899)	(0.0752, 0.3236 )							(0.8967)		
ESE	1.1164	-	0.0639	-	-	−413.915	831.830	837.535	831.926	0.0786	0.8107	0.1365
	(0.8549, 1.3779)		(0.0504, 0.0774)							(0.4070)		
SW	-	0.9920	0.0611	-	-	−414.325	832.650	838.354	832.746	0.0699	0.8312	0.1400
		(0.8644, 1.1196)	(0.0377, 0.0844)							(0.5581)		
W	-	1.0476	0.0939	-	-	−414.087	832.174	837.878	832.270	0.0699	0.7815	0.1308
		(0.9152, 1.1800)	(0.0565, 0.1314)							(0.5123)		
GR	0.0476	-	-	0.3641	-	−429.225	862.450	808.154	862.546	0.1551	2.7699	0.4729
	(0.9255, 1.5082)			(0.0945, 0.1477)						(0.0042)		
GIW	0.1988	0.7521	-	-	8.1915	−444.000	894.002	902.558	894.195	0.1408	4.513	0.7414
	(0, 0.6116)	(0.6689, 0.8352)			(0, 20.8093)					(0.00125)		
HLP	0.0555	-	4.0202	-	-	−413.171	830.342	836.046	830.438	0.0954	0.3620	0.0606
	(0, 0.1237)		(0, 9.4418)							(0.1861)		

**Table 4:** MLEs, $L_{R}$, R, KS with *p*-value in parenthesis, and 95% CI with confidence length below for stress strength data sets.

$\alpha_{1}$	$\alpha_{2}$	$\beta_{1}$	$\beta_{2}$	λ	$L_{R}$	${\mathrm{KS}}_{1}$	${\mathrm{KS}}_{2}$	R	${\mathrm{CI}}_{\mathrm{Bp}}$	${\mathrm{CI}}_{\mathrm{Bt}}$
5.9420	3.7152	2.3651	2.7151	0.0877	106.265	0.0946	0.0485	0.7837	(0.7073, 0.8524)	(0.7073, 0.8602)
						(0.5921)	(0.9944)		0.1451	0.1529

## Data Availability

Not applicable.
